# An Integrated Computational/Experimental Model of Lymphoma Growth

**DOI:** 10.1371/journal.pcbi.1003008

**Published:** 2013-03-28

**Authors:** Hermann B. Frieboes, Bryan R. Smith, Yao-Li Chuang, Ken Ito, Allison M. Roettgers, Sanjiv S. Gambhir, Vittorio Cristini

**Affiliations:** 1 Department of Bioengineering, University of Louisville, Louisville, Kentucky, United States of America; 2 James Graham Brown Cancer Center, University of Louisville, Louisville, Kentucky, United States of America; 3 Department of Pathology, University of New Mexico, Albuquerque, New Mexico, United States of America; 4 Molecular Imaging Program at Stanford (MIPS), Department of Radiology, Stanford University, Stanford, California, United States of America; 5 Bioengineering, Materials Science & Engineering, Bio-X, Stanford University, Stanford, California, United States of America; 6 Department of Chemical Engineering, University of New Mexico, Albuquerque, New Mexico, United States of America; University of Notre Dame, United States of America

## Abstract

Non-Hodgkin's lymphoma is a disseminated, highly malignant cancer, with resistance to drug treatment based on molecular- and tissue-scale characteristics that are intricately linked. A critical element of molecular resistance has been traced to the loss of functionality in proteins such as the tumor suppressor *p53*. We investigate the tissue-scale physiologic effects of this loss by integrating *in vivo* and immunohistological data with computational modeling to study the spatiotemporal physical dynamics of lymphoma growth. We compare between drug-sensitive *Eμ-myc Arf-/-* and drug-resistant *Eμ-myc p53-/-* lymphoma cell tumors grown in live mice. Initial values for the model parameters are obtained in part by extracting values from the cellular-scale from whole-tumor histological staining of the tumor-infiltrated inguinal lymph node *in vivo*. We compare model-predicted tumor growth with that observed from intravital microscopy and macroscopic imaging *in vivo*, finding that the model is able to accurately predict lymphoma growth. A critical physical mechanism underlying drug-resistant phenotypes may be that the *Eμ-myc p53-/-* cells seem to pack more closely within the tumor than the *Eμ-myc Arf-/-* cells, thus possibly exacerbating diffusion gradients of oxygen, leading to cell quiescence and hence resistance to cell-cycle specific drugs. Tighter cell packing could also maintain steeper gradients of drug and lead to insufficient toxicity. The transport phenomena within the lymphoma may thus contribute in nontrivial, complex ways to the difference in drug sensitivity between *Eμ-myc Arf-/-* and *Eμ-myc p53-/-* tumors, beyond what might be solely expected from loss of functionality at the molecular scale. We conclude that computational modeling tightly integrated with experimental data gives insight into the dynamics of Non-Hodgkin's lymphoma and provides a platform to generate confirmable predictions of tumor growth.

## Author Summary

Non-Hodgkin's lymphoma is a cancer that develops from white blood cells called lymphocytes in the immune system, whose role is to fight disease throughout the body. This cancer can spread throughout the whole body and be very lethal – in the US, one third of patients will die from this disease within five years of diagnosis. Chemotherapy is a usual treatment for lymphoma, but the cancer can become highly resistant to it. One reason is that a critical gene called *p53* can become mutated and help the cancer to survive. In this work we investigate how cells with this mutation affect the cancer growth by performing experiments in mice and using a computer model. By inputting the model parameters based on data from the experiments, we are able to accurately predict the growth of the tumor as compared to tumor measurements in living mice. We conclude that computational modeling integrated with experimental data gives insight into the dynamics of Non-Hodgkin's lymphoma, and provides a platform to generate confirmable predictions of tumor growth.

## Introduction

Monoclonal antibodies and small molecule inhibitors of intracellular targets are being developed alongside a host of anti-non-Hodgkin's lymphoma therapeutic options [1]. Yet the tumor tissue-scale effects from these molecular-scale manipulations are not well-understood. With the ultimate goal to more rationally optimize lymphoma treatment, we integrate pre-clinical *in vivo* observations of lymphoma growth with computational modeling to create a platform that could lead to optimized therapy. As a first step towards this goal, we develop the capability for simulation in order to gain insight into the tissue-scale effect of molecular-scale mechanisms that drive lymphoma growth. We use the modeling to study these mechanisms and their association to cell proliferation, death, and physical transport barriers within the tumor tissue.

Tumor growth and treatment response have been modeled using mathematics and numerical simulation for the past several decades (see recent reviews [2]–[9]). Models are usually either discrete or continuum depending on how the tumor tissue is represented. *Discrete models* represent individual cells according to a specific set of bio-physical and -chemical rules, which is particularly useful for studying carcinogenesis, natural selection, genetic instability, and cell-cell and cell-microenvironment interaction (see reviews by [10]–[20]). *Continuum models* treat tumors as a collection of tissue, applying principles from continuum mechanics to describe cancer-related variables (e.g., cell volume fractions and concentrations of oxygen and nutrients) as continuous fields by means of partial differential and integro-differential equations [2]. A third modeling approach employs a *hybrid* combination of both continuum and discrete representations of tumor cells and microenvironment components, aiming to develop multiscale models where the discrete scale can be directly fitted to molecular and cell-scale data and then upscaled to inform the phenomenological parameters at the continuum scale (see recent work by [21]–[23]).

There is a paucity of mathematical oncology work applied to the study of non-Hodgkin's lymphoma, with some notable exceptions providing insight into the role of the tumor microenvironment heterogeneity in the treatment response [24], [25] and the disease origin [26]. Like many other cancers (solid tumors), two critical tissue-scale effects in lymphoma are hypoxia and angiogenesis, as observed in our studies and other work [27]. Supporting previous qualitative observations of physiological resistance, mathematical modeling and computational simulation have shown that the diffusion barrier alone can result in poor tumor response to chemotherapy due to diminished delivery of drug, oxygen, and cell nutrients [28], [29]. Local depletion of oxygen and cell nutrients may further promote survival to cell cycle-specific drugs through cell quiescence.

In order to study these effects in lymphoma, we implement an integrated computational/experimental approach to quantitatively link the processes from the cell scale to the tumor tissue-scale behavior in order to gain insight into their cause and progression in time. We extend a version of our 3D continuum model [30]–[32], building upon extensive mathematical oncology work [2], [3], [33]–[35], and calibrate both parameters and equations, i.e., functional relationships that are not conservation laws, from detailed experimental data to produce a virtual lymphoma. We obtain the experimental data by very fine sectioning of both drug-sensitive and -resistant lymphomas, thus visualizing molecular, cellular, and tissue-scale parameter information across the whole tumor geometry. We further develop the protocols for calibration of parameters by building on recent work based on patient histopathology [36], [37]. We also use the data to derive the relationships between model parameters for apoptosis, proliferation, and vasculature. We verify the model results at the tumor-scale through tissue-scale observations *in vivo* of tumor size, morphology, and vasculature using intravital microscopy and macroscopic imaging of the inguinal lymph node. We note that comparison of model results to experimental data has been done to various extents for different cancers (see reviews above); here, we perform a tissue-scale comparison after extensive calibration of cell-scale parameters in order to validate the model results. We undertake simulations to study how the growth of drug-resistant Non-Hodgkin's lymphoma may be governed by the cellular phenotype, and use this information to better elucidate the links between physical drug resistance and molecular-scale phenotype by experimental and computational comparison to drug-sensitive tumors.

This process yields a lymphoma simulator as an initial step to study detailed tumor progression and provide further insight into drug resistance, and, ultimately, may provide a tool to design better personalized treatments for Non-Hodgkin's lymphoma. Since the cell-scale measurements used for calibration are different from those at the tissue-scale used for verification, this methodology enables the model to bridge from the cell to the tumor scale to calculate tumor growth and hypothesize associated mechanisms predictively, i.e., without resorting to fitting to the experimental data. This process quantitatively links the cellular phenotype to the tumor tissue-scale behavior, and may serve to highlight the importance of physical heterogeneity and interactions in the tumor microenvironment when evaluating chemotherapeutic agents in addition to consideration of chemo-protective effects such as cell-specific phenotypic properties and cell-cell and cell-ECM adhesion [38].

## Materials and Methods

### Experimental model

We choose an *Eμ-myc* murine orthotopic lymphoma experimental model because of its similarity to human Non-Hodgkin's Lymphoma [39], and select five parameters to measure based on their importance to lymphoma progression: viability, hypoxia, vascularization, proliferation, and apoptosis. In order to investigate the role of physical heterogeneity in the development of drug resistance, including the impediment of transport barriers, we focus on two types of lymphoma cells: *Eμ-myc Arf-/-* cells (Doxorubicin (DOX) and Cyclophosphamide (CTX) sensitive, with IC_50_ = 3.5 nM and 16.0 µM, respectively; the IC_50_ is the amount of drug needed to kill 50% of a cell population), and *Eμ-myc p53-/-* cells (DOX and CTX resistant: IC_50_ = 46.2 nM and 75.8 µM, respectively). The *Eμ-myc* transgenic mouse model expresses the *Myc* oncogene in the B cell compartment, resulting in mice with transplantable B cell lymphomas. We chose this *in vivo* model because it captures genetic and pathological features of the human disease and, given the appropriate genetic mutation, drug-resistant and drug-sensitive tumors can be directly compared [39],[40].

### Cell culture

*Eμ-myc/Arf-/-* and *Eμ-myc/p53-/-* lymphoma cells, which harbor loss-of-function regions in the *Arf* and *p53* genes respectively, were previously derived by intercrossing *Eμ-myc* transgenic mice with *Arf*-null and *p53*-null mice, all in the C57BL/6 background as described previously [39]. *Eμ-myc/Arf-/-* lymphoma cells and *Eμ-myc/p53-/-* lymphoma cells were cultured in 45% Dulbecco's modified Eagle medium (DMEM) and 45% Iscove's Modified Dulbecco's Medium (IMDM) with 10% fetal bovine serum (FBS) and 1% penicillin G-streptomycin onto the feeder cells – Mouse Embryonic Fibroblasts (MEFs).

### Murine lymphoma model

C57BL/6 mice were obtained from Charles River Laboratories (Wilmington, Massachusetts). All animal studies were approved by The Stanford University Institutional Animal Care and Use Committee. Lymphoma cells (1×10^6^) *Eμ-myc/Arf-/-* and *Eμ-myc/p53-/-* were diluted with 200 µl of PBS and injected intravenously via the tail vein as described previously [39]. The intravital microscopy and macroscopic tumor observations were obtained for at least n = 4 mice per tumor group.

### Immunohistochemistry

**Figure 1:**
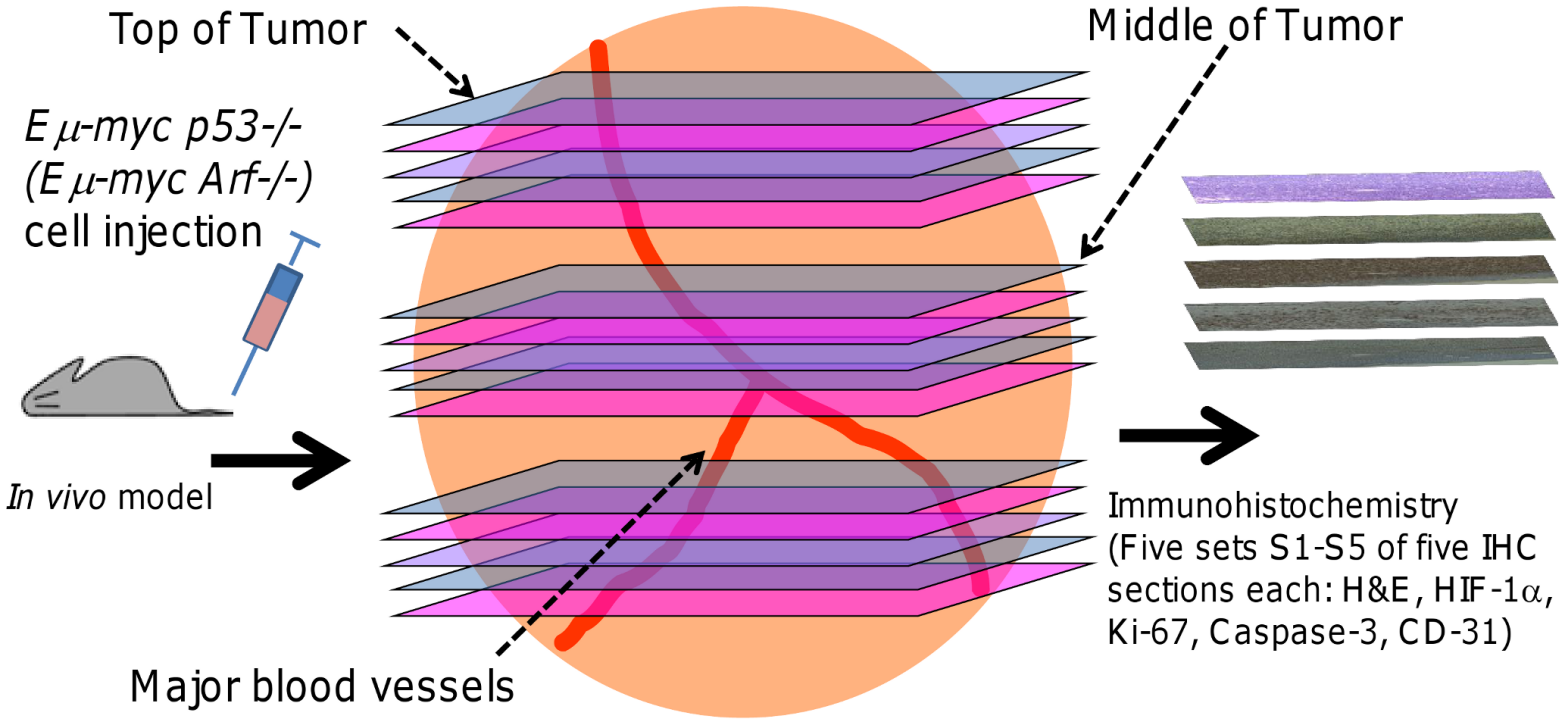
Scheme to obtain the cellular-scale experimental data. Lymphomas (shown as large orange sphere) were grown *in vivo* by tail vein injection of either drug-sensitive *Eμ-myc/Arf-/-* or drug-resistant *Eμ-myc/p53-/-* lymphoma cells. The inguinal lymph node tumor was excised, fixed, and sliced for histology sections (5 µm apart) every 100 µm along the tumor. A total of five sets (S1 through S5) of histology sections were obtained (for simplicity, the figure only shows three sets). The sections in each set were stained for cell viability (H&E), hypoxia (HIF-1α), proliferation (Ki-67), apoptosis (Caspase-3), and vascularization (CD-31).

We isolated both *Eμ-myc/Arf-/-* and *Eμ-myc/p53-/-* driven tumors at day 21 after tail-vein injection of lymphoma cells. Typical murine lymphomas were observed to range from about 4 to 6 mm in diameter prior to fixation. Lymph node tissues were fixed and paraffin-embedded. The tissues were used for immunohistochemical (IHC) identification of cell viability (H&E staining), hypoxia (HIF-1α), vascularization (CD31), proliferation (Ki-67), and apoptosis (Caspase-3). Five 2-µm thick sections were cut 5 µm apart from each other in order to stain for these markers (**Figure 1**). A total of five sets (S1 through S5) of five stained sections each was collected every 100 µm along the lymphoma, in order to section and stain the entire tumor for sequential microscopic scanning of the stained sections. Sections S1 and S5 were at the tumor top and bottom, respectively, while the other sections were towards the center with S3 being in the middle. Note that due to tissue processing and dehydration, the tumors as cut were smaller than measured when removed from the animal. All the sections were de-paraffinized and rehydrated in PBS. Then the sections in each set were incubated at 4°C with the primary antibody overnight: rabbit anti-mouse HIF-1 antibody (Abcam, Santa Cruz, CA), rabbit anti-mouse Ki-67 antibody (Labvision, Fremont, CA), rabbit anti-mouse Caspase-3 antibody (Cell Signaling Technology, Beverly, CA), and rat anti-mouse CD31 antibody (BD Pharmingen, San Diego, CA), and incubated for 1 hour at room temperature with a peroxidase-conjugated secondary antibody. The samples were fully scanned and stitched together using a digital pathology BioImagene instrument (Ventana Medical Systems, Tucson AZ) at ×20 magnification.

### Mathematical model

**Figure 2:**
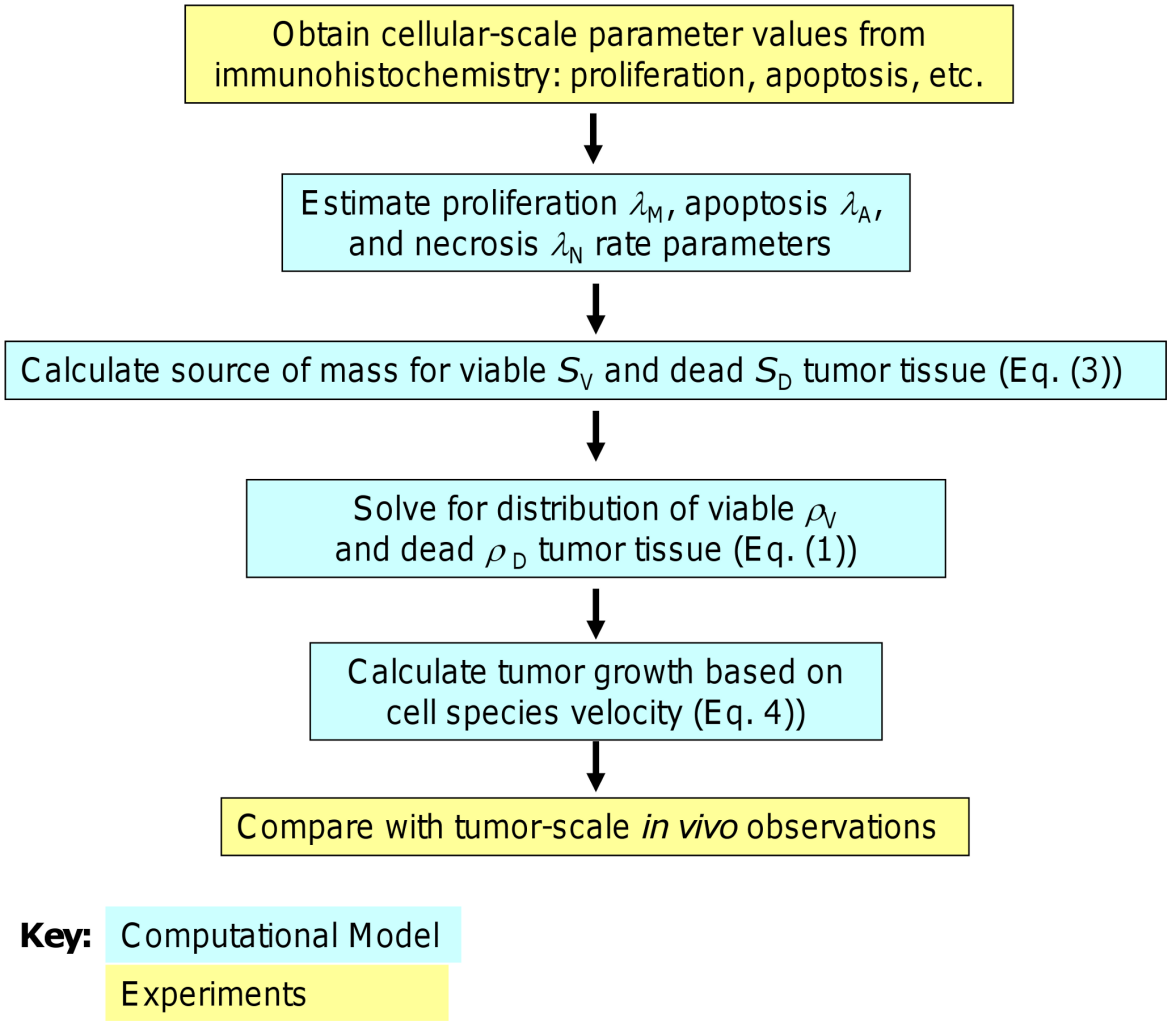
Algorithm flowchart. Refer to **Materials and Methods** and **Text S1** for equations. Using the cellular-scale data, we measured values for proliferation and apoptosis for both drug-sensitive and drug-resistant tumors and calculated corresponding values for the model mitosis and apoptosis parameters *λ*_M_ and *λ*_A_. We solved Eq. (2) for the local levels of cell substrates *n* at each time step of simulation of tumor growth. The parameters were input into Eq. (3) to numerically calculate the source mass terms *S_i_*, which were then used in Eq. (1) to compute the volume fractions of viable *ρ*_V_ and *ρ*_D_ dead tissue. These fractions were used in Eq. (4) to obtain the tumor tissue growth velocity.

**Figure 3:**
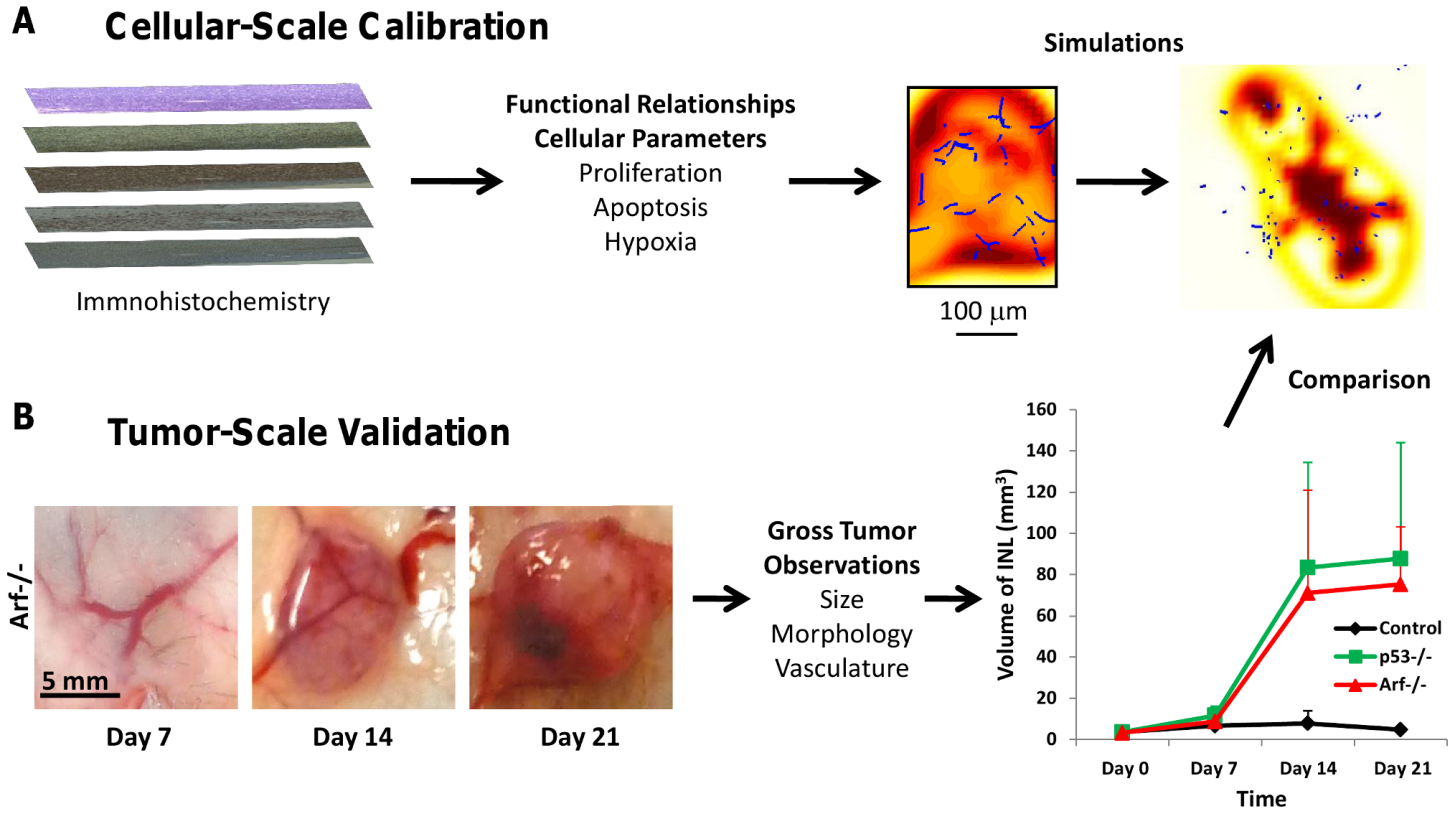
Schematic showing integrated computational/experimental modeling strategy involving both cell- and tumor-scale measurements. (**A**) Functional relationships involving cell-scale parameters such as proliferation (Ki-67), apoptosis (Caspase-3), and hypoxia (HIF-1α) are defined based on experimental observations, e.g., from immunohistochemistry the density of viable tissue as a function of vascularization is shown in the third panel (red: highest density; yellow: lowest; blue: vessels). These functional relationships as well as parameter values measured experimentally are then used as input to the model to create simulations of lymphoma growth. A sample simulated tumor cross-section showing vascularized viable tissue (highest density in red, lowest in yellow, with vessel cross-sections as small blue dots) is shown at the far right. (**B**) Lymphoma observations regarding size, morphology, and vasculature from macroscopic imaging of an inguinal lymph node in live mice provide part of the tumor-scale information to validate the model simulations. Note the pre-existing vasculature in the lymph node (in the center of each frame) from which oxygen and nutrients are supplied to the tissue. For comparison, a control group of lymph nodes in animals without tumors is also shown.

The model treats tissue as a mixture of various cell species, water, and ECM; each component is subject to physical conservation laws described by diffusion-taxis-reaction equations (see below). Briefly, the tissue microstructure is modeled through the proper choice of parameter values and through biologically-justified functional relationships between these parameters, e.g., cellular transitions from quiescence to proliferation depend upon oxygen concentration [41]. The model simulates non-symmetric tumor evolution in 2D and 3D, and dynamically couples heterogeneous growth, vascularization, and tissue biomechanics (**Figure 2**). In [36] we calibrated models using cell-scale data to predict tissue scale parameters such as size and growth rate. These models are predictive because they are not calibrated with the same data used for model validation, which avoids data fitting. While in [36] we focused on the final predicted tumor sizes, here we focus on the growth rate as an essential first step; in follow-up work, we will evaluate the complex problem of drug response. Our approach to constrain the computational model involves both cell- and tumor-scale approaches as described in **Figure 3**.

**Figure 4:**
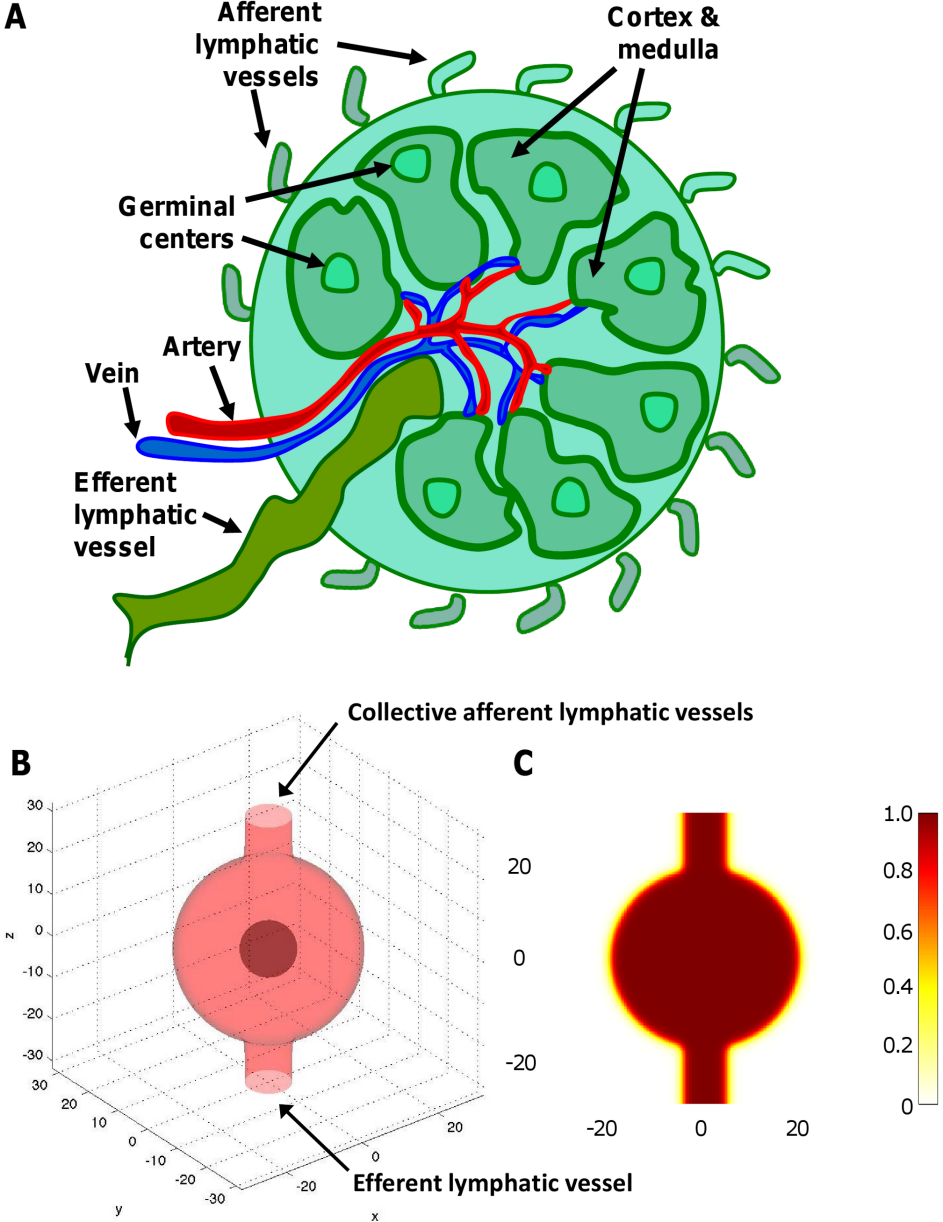
Representation of the lymph node by the computational model. (A) Diagram highlighting a typical lymph node structure. (B) Simulation output from the model showing an incipient tumor (dark red) forming in the center of the node. Afferent lymphatic vessels are collectively represented as one incoming tube on the top, and the efferent vessel is at the bottom. (C) The simulated distribution of oxygen (brown color) released by the blood vasculature within the node remains uniform at this initial stage.

We approximate the healthy lymph node as a sphere to represent the experiments in the mouse model (**Figure 4**). To simulate node expansion and deformation of surrounding tissue to accommodate the growing tumor, as a first step we delineate the tumor boundary by decreasing the value of the cell mobility parameter beyond the sphere diameter (see below). For the multigrid algorithm, we pick a computational domain that is a 6.4 mm× 6.4 mm× 6.4 mm box, with finest mesh grid size = 100 microns; this grid size provides adequate resolution to resolve the tumor boundaries without incurring excessive computational cost.

### Distribution of cell species

We assume that the tumor is a mixture of cells, interstitial fluid, and extracellular matrix (ECM). The temporal rate of change in viable and dead tumor tissue at any location within the tumor equals the amount of mass that is pushed, transported, and pulled due to cell motion, adhesion, and tissue pressure, plus the net result of production and destruction of mass due to cell proliferation and death:
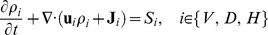
(1)The rate of change in the volume fraction *ρ_i_* of cell species *i* (*V*: viable tumor; *D*: dead tumor; *H*: host) is specified throughout the computational domain by balancing net creation (*S_i_*: proliferation minus apoptosis and necrosis; see below) with cell advection (∇·(**u** *_i_ρ_i_*), where **u** *_i_* is the velocity of the cell species) and cell-cell and cell-ECM interactions (adhesion, cell incompressibility, chemotaxis, and haptotaxis, incorporated in a flux **J** *_i_*) [31], [32]. The reticular network within the lymph node contains a variety of extracellular matrix proteins, many of which are known ligands for integrin cell surface adhesion receptors [42], [43]. Cell-cell and cell-ECM mechanical interactions are modeled through **J** using a generalized Fick's Law [31].

### Angiogenesis

Tumor angiogenesis is driven by excessive accumulation of cancerous cells, leading to a chronic under-supply of oxygen and cell nutrients (generically here labeled “nutrients”) in tumor regions farther removed from pre-existing vessels [44]. Hypoxic cells in lymphoma release a net balance of pro-angiogenic factors such as VEGF-A, bFGF, PDGF and VEGF-C, which promote neo-vascularization mainly through sprouting angiogenesis of mature resident endothelial cells and, to a lesser extent, through vasculogenesis from recruitment of bone marrow-derived progenitor cells [45]. Accordingly, the model incorporates angiogenesis into the lymphoma by coupling with a multiscale representation of tumor vessel growth, branching, and anastomosis based on earlier work [46]–[48] (further details in **Text S1**).

### Transport

The vasculature releases oxygen and nutrients *n* that diffuse through the tissue and are uptaken by cells during metabolism, while tumor cells secrete VEGF (*n_V_*) in response to hypoxia [32]. The oxygen and nutrients are non-dimensionalized by the maximum inside vessels, hence their levels are ≤1 and are assumed to be stationary. The transport can be described as:
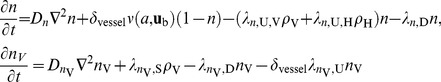
(2)where *D_n_* and 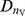 are diffusion constants (1×10^−5^ cm^2^/sec for oxygen [49] and 1×10^−7^ cm^2^/sec for VEGF [50]), *δ*_vessel_ (Dirac delta function) is the indicator function of vasculature (1 where it exists and 0 otherwise), *ν* is the delivery rate (depends upon *a*, capillary vessel cross-sectional area, and **u**_b_, blood velocity), 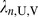, 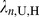, and 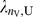 are the uptake rates, 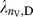 and 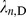 are the decay rates (for simplicity, assumed to be zero), and 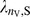 is the secretion rate.

### Proliferation, apoptosis, and necrosis

The tumor species viable (*V*) volume fraction *ρ_V_* is assumed to increase through proliferation and decrease through apoptosis and necrosis. We assume that normal host cells (*H*) do not proliferate, but may also undergo apoptosis (*A*) and necrosis (*N*); the total volume fraction of dead cells (*D*) is *ρ_D_*. For simplicity, we assume these primarily affect tumor mass through the transport of water within the tissue and hence neglect their solid fraction. Under the assumption that a dense viable cell population prevents nutrient saturation, we model the proliferation as directly proportional to (non-dimensionalized) nutrient substrate *n* above a threshold level *n*_N_, resulting in the net creation of one cell by removing the equivalent water volume from the interstitium. Cells experiencing a substrate level below *n*_N_ are considered quiescent (e.g., due to hypoxia). Apoptosis transfers cells from the viable tumor and host cell species to the dead cell species, where cells degrade and release their water content; this models phagocytosis of apoptotic bodies by neighboring viable cells and the subsequent release of the water of lysed cells. Necrosis occurs when the nutrient substrate concentration falls below the threshold *n_N_* and ultimately releases the cellular water content (i.e., we assume that the main mode of cell death due to lack of nutrients is mainly represented by necrosis). The resulting model is:
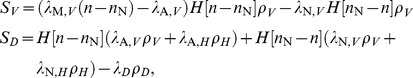
(3)where *λ*_M,*i*_, *λ*_A,*i*_, and *λ*_N,*i*_ are mitosis, apoptosis, and necrosis rates, *λ*_D_ is the cell degradation rate (varies due to the differences between apoptosis and necrosis), and *H*(*x*) is the Heaviside “switch” function.

### Velocity of cell species

The movement of a cell species is determined by the balance of proliferation-generated oncotic pressure, cell-cell and cell-ECM adhesion, as well as chemotaxis (due to substrate gradients), and haptotaxis (due to gradients in the ECM density). We model the motion of cells and interstitial fluid through the ECM as a viscous, inertialess flow through a porous medium. Therefore, no distinction between interstitial fluid hydrostatic pressure and mechanical pressure due to cell-cell interactions is made. Cell velocity is a function of cell mobility and tissue oncotic (solid) pressure (Darcy's law); cell-cell adhesion is modeled using an energy approach from continuum thermodynamics (see **Text S1**). For simplicity, the interstitial fluid is modeled as moving freely through the ECM (i.e., at a faster time scale than the cells).
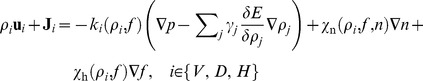
(4)The variational derivative δ*E*/δ*ρ_i_* of the cell-cell interaction potential, combined with the remaining contributions to the flux **J** (due to pressure, haptotaxis, and chemotaxis; see **Text S1**), yields a generalized Darcy-type constitutive law for the cell velocity **u**_i_ of a cell species *i*, determined by the balance of proliferation-generated oncotic pressure *p*, cell-cell and cell-ECM adhesion, as well as chemotaxis (due to gradients in the cell substrates *n*), and haptotaxis (due to gradients in the ECM density *f*) [32]. *k_i_* is cellular mobility, reflecting the response to pressure gradients and cell-cell interactions, *γ_j_* is the adhesion force, and *χ_n_* and *χ_h_* are the chemotaxis and haptotaxis coefficients, respectively (see **Table S1**). For the host cells, *χ*_n_ = *χ*_h_ = 0. The Supplemental **Text S1** further describes the ECM density *f* as well as the effect of the cell velocity on the lymph node geometry.

## Results

### Comparison between *Eμ-myc p53-/-* and *Eμ-myc Arf-/-* tumors

**Figure 5:**
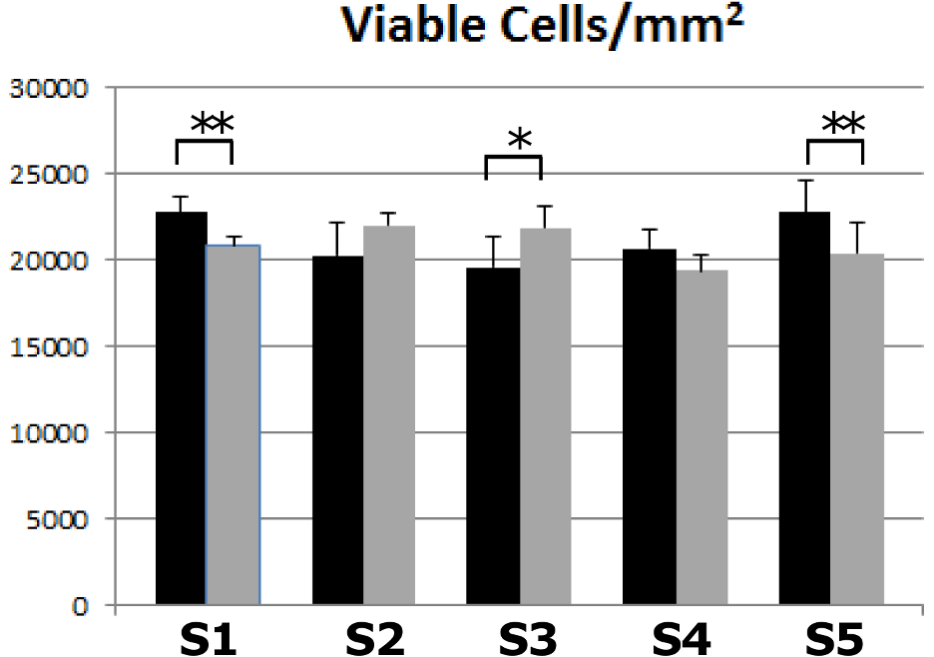
Lymphoma tumor cell viability. Viability per area was measured along the five sets (S1 through S5) of histology sections for *Eμ-myc Arf-/-* (black) and *Eμ-myc p53-/-* (gray) tumors. All error bars represent standard deviation from at least n = 3 measurements in each section. Asterisks show level of statistical significance determined by Student's t-test with α = 0.05 (one asterisk, p<0.05; two asterisks, p<0.01).

We used the IHC staining to estimate the number and spatial localization of cells that were viable (from H&E), proliferating (from Ki-67), apoptotic (from Caspase-3), hypoxic (from HIF-1α), and with vascular endothelial characteristics (from CD31). These estimates were calculated for both *Eμ-myc Arf-/-* and *Eμ-myc p53-/-* cells for each set of five sections obtained every 100 µm across the lymphoma (**Figures 5** **and** **6**).

**Figure 6:**
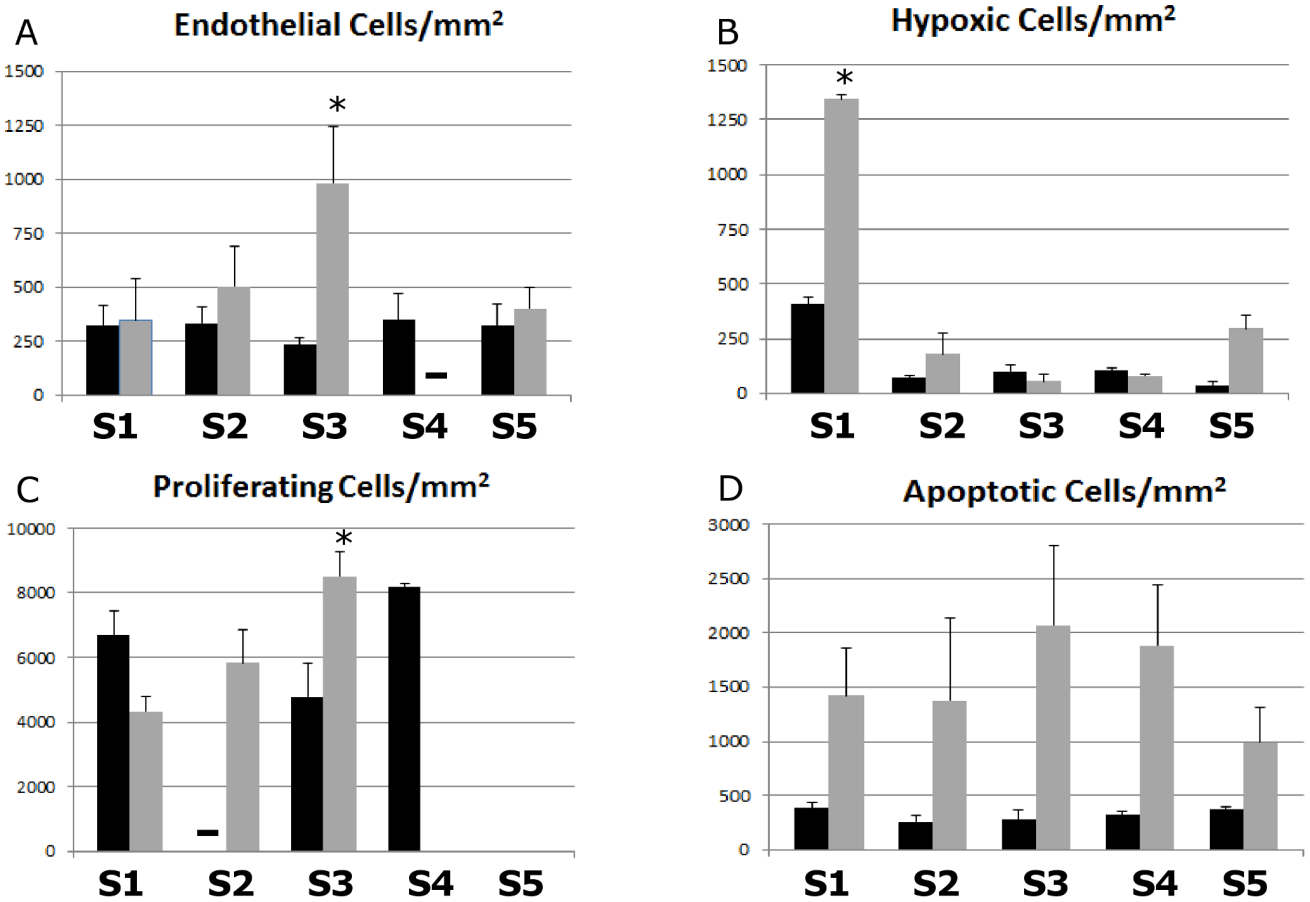
Lymphoma tumor characteristics. Histological measurements are shown *Eμ-myc Arf-/-* (black) and *Eμ-myc p53-/-* (gray) tumors along the five sets of sections (S1 through S5) of the lymphoma: (A) Endothelial cells per area; (B) hypoxic cells per area; (C) proliferating cells per area; (D) apoptotic cells per area. Sections S1 and S5 are at the tumor top and bottom, respectively, while the other sections are in the interior with S3 being in the middle. Dashes in panels (A) and (C) indicate that no data was obtained; in panel (C), no proliferation was detected for *Eμ-myc p53-/-* cells in sets S4 and S5, and none for *Eμ-myc Arf-/-* in set S5, probably due to sample defects. All error bars represent standard deviation from at least n = 3 measurements in each section; asterisk indicates statistical significance (p<0.05) determined by Student's t-test with α = 0.05. The data shows that for *Eμ-myc p53-/-* there is higher vascularization in the center, higher hypoxic density on the periphery, and higher overall apoptotic density compared to *Eμ-myc Arf-/-*.

A comparison of viable *Eμ-myc p53-/-* to *Eμ-myc Arf-/-* cells along the lymphoma (**Figure 5**) indicates that the viability is higher for the drug-resistant tumors in the middle of the tumor (Section S3) compared to the drug-sensitive tumors, with a corresponding statistically significant increase in cell density (p = 0.024; Student's t-test with α = 0.05). In contrast to the *Eμ-myc p53-/-* tumors, the *Eμ-myc Arf-/-* seemed to be more dense in the peripheral regions (p = 0.002 on one end (Section S1) and p = 0.009 on the other end (Section S5)), whereas they were about the same for both tumor types in the intermediate sections S2 and S4. Tumors with drug-resistant cells have a 4-fold increase in endothelial cells in the core of the tumor (Section S3) compared to drug-sensitive tumors (**Figure 6A**). Hypoxia is higher in the peripheral regions for the *Eμ-myc p53-/-* (**Figure 6B**) even though for both tumor types the peripheral regions seem to be equally vascularized (based on the endothelial cell density). This could be due to the vasculature on the periphery not being fully functional, with a potential difference in vascular function between the two tumor types leading to a more hypoxic phenotype for the *Eμ-myc p53-/-*. Although the core proportionally holds almost twice the number of proliferating cells for the drug-resistant tumors as compared to the drug-sensitive case (**Figure 6C**), a correlation between proliferation and vascularization/hypoxia is precluded. Interestingly, the number of apoptotic cells is consistently higher for *Eμ-myc p53-/-* (**Figure 6D**), suggesting non-hypoxia driven apoptosis for these tumors.

### Model calibration with cellular-scale data

By analyzing each IHC section longitudinally along the tumor, a range of baseline values can be calculated from the experimental data for key model parameters (**Table S1**), inspired by recent methods in mathematical pathology [36]: cell viability, necrosis, and spatial distribution pattern (from H&E), cell proliferation (from Ki-67), cell apoptosis (from Caspase-3), oxygen diffusion distance (from HIF-1α), and blood vessel density (from CD31). These values are obtained for both *Eμ-myc Arf-/-* and *Eμ-myc p53-/-* tumors for each of the five sections obtained longitudinally along the tumor, with values sampled from the middle (core) and the edge (periphery) of each section. The measured values are not resolved in space but averaged over each section, thus yielding information averaged over space. The periphery was defined as the region approximately within 200 µm of the tumor boundary.

**Figure S1** shows an example of this calibration process for proliferation at the periphery and middle from two histology sections in the center of the tumor (Section S3). Taking an average proliferation cycle of 20 hours that we observed for the lymphoma cells in culture, the proliferation calculation in units of day^−1^ is λ_M_*<n> = [(stained/(stained+unstained))/20 hours/prolif.] * 24 hours/day. The average nutrient *<n>* indicates that this proliferation rate depends on the model diffusion of cell substrates such as glucose and oxygen in the 3D space (**Eq. 2**). Similarly, since the apoptosis cycle was detectable up to 5 hours, the apoptosis calculation in units of day^−1^ is λ_A_ = [(stained/(stained+unstained))/5 hours/apoptosis] * 24 hours/day.

We calculate the average nutrient from the blood vessel density by assuming a uniform nutrient delivery rate from the blood to the tissue adjacent to the vessels (**Eq. 2**). Estimating blood vessel area versus surrounding tissue provides a measure of the magnitude of cell substrates transferred into the tumor. Thus, we calculate the fraction of cells supported per endothelial cell in a unit volume to be (number unstained/(number stained+unstained))^3/2^. When the viable cell fraction in the simulations matches what is directly observed from microscopy, this implies that the vascular and nutrient distributions have been correctly represented in the model (**Figure 3A** **, middle**). Similarly, we calculate the hypoxic cell fraction per unit volume as (number stained/(number stained+unstained))^3/2^.

### Modeling of the lymph node

The node is represented by the computational model initially as a spherical capsule in 3D with a membrane boundary separating it from the surrounding tissue (**Figure 4**) (see **Text S1**). Lymphoma cells are assumed to enter the lymph node through the afferent lymph vessels. As they accumulate in the node during tumor progression in time, they compete for cell substrates such as oxygen and nutrients with the normal lymphocytes. These substrates are assumed to diffuse radially outward toward the node periphery from the pre-existing vasculature, situated mainly in the core of the node (see **Figure 4A** and **Figure 3B** **, left**, at the intersection of three large blood vessels). Once a tumor has begun to form in the core of the node, this diffusion process presents a transport barrier for oxygen and nutrients to the lymphoma cells incoming through the afferent vessels into the node.

### Assessment of the model

We investigate the effect of initially available oxygen and cell substrates needed for cell proliferation, since lymphoma growth is hypothesized to depend on access to these through the vasculature. Preliminary calculations suggested that the initially available nutrient level has a significant effect on the growth phase of the tumor but not on its terminal size, which according to a theoretical analysis of the model (**Text S1**) depends mainly on the ratio of apoptosis to proliferation [51]. A further investigation revealed that the initial guess of parameter values results in a mismatch between the ratio of hypoxic cells and the average apoptosis rate: where the range of hypoxic ratio matches the experiments, the apoptosis rate range in the model is too low.

Accordingly, we calibrated the cell necrosis rate so that the key parameter values remain invariant when the initial nutrient is set to a threshold of 0.5. With this set of parameters, a necrosis rate from 5 to 7 (non-dimensional units) would satisfy the experimentally observed ranges of both the hypoxic fractions and the average apoptosis rate (**Figure S2A–B**). We then varied the initial nutrient threshold while maintaining the necrosis rate invariant to confirm that the fraction of hypoxic cells and average apoptosis rate would remain within the experimentally observed range of values (**Figure S2C–D**). This calibration suggests that the initially available nutrient still affects the growth phase of lymphoma. In this model, the lymphoma tumor and the lymph node greatly outgrow the original lymph node size, which we consistently observed *in vivo* in addition to the distortion of the lymph node geometry (we are currently implementing the Diffuse Domain Method [52] to better represent this geometry).

### Prediction of lymphoma growth

**Figure 7:**
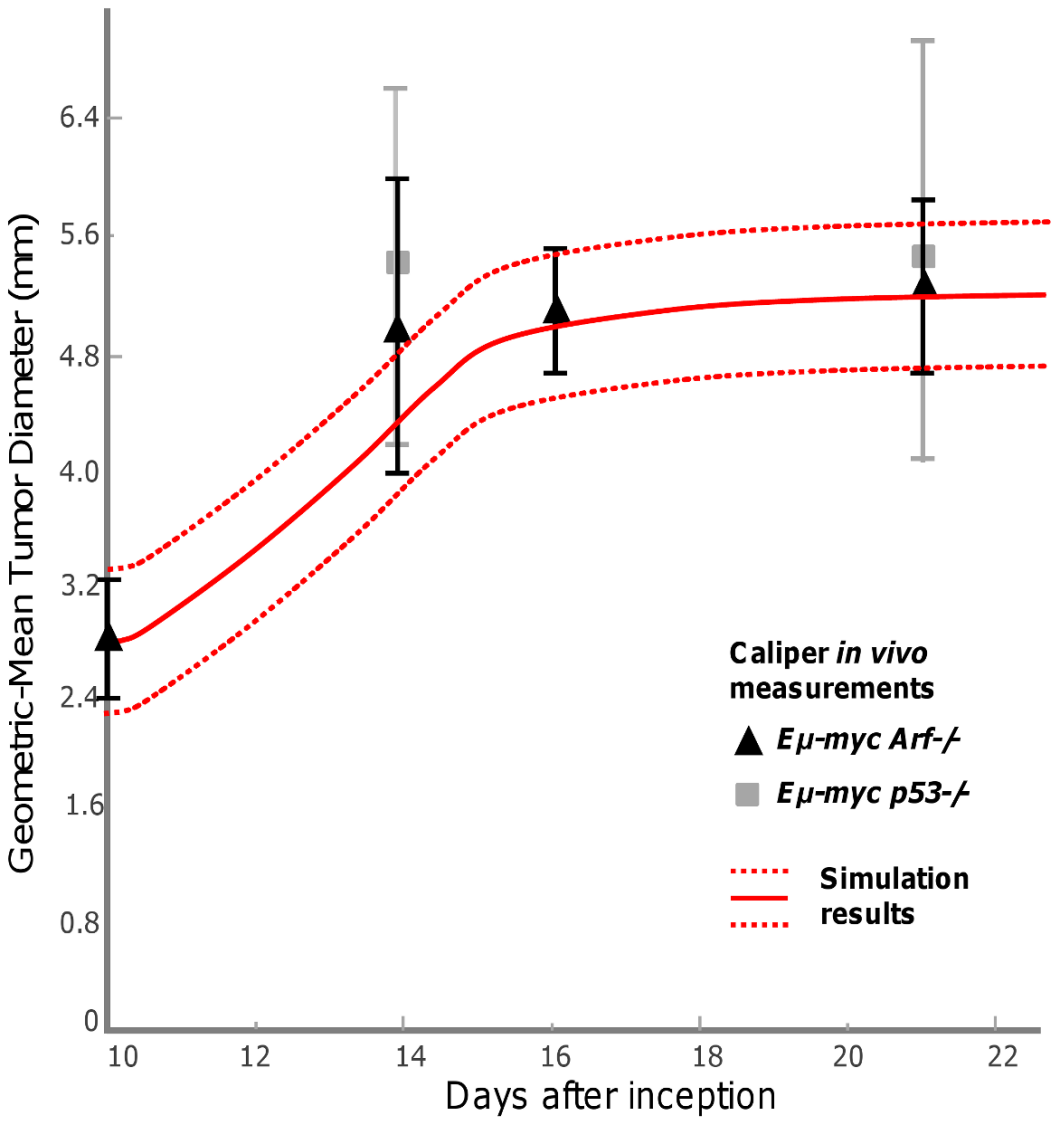
Prediction of lymphoma growth based on the calibrated model parameters. Simulated mean tumor diameter (solid red line) bounded by variation in the measured oxygen diffusion distance (dashed red lines) falls within the range of values measured for the tumor growth observed *in vivo* (denoted by the triangles and squares with vertical error bars). Note that the simulated growth is the same for both *Eμ-myc Arf-/-* and *Eμ-myc p53-/-* tumors.

After using the IHC data to perform a cell-scale calibration of the lymphoma model, we verify the simulated tissue-scale lymphoma size from *in vivo* macroscopic observations and intravital imaging at the tissue scale. Recently, it has been discovered with bioluminescence imaging by Gambhir and co-workers that lymphoma cells coming from the spleen and bone marrow seed the inguinal lymph node around Day 9 *in vivo* [53]. Using this seeding as the initial condition for the simulations, the model predicts the tumor diameter to be ∼5.2±0.5 mm by Day 21 (**Figure 7**). This figure also shows the gross tumor size from our caliper measurements in time, indicating that the model-predicted tumor diameter for the maximum possible value of initial nutrient falls within the range of the measurements *in vivo* (the experiments show that there is no statistical difference in the tumor growth between the two cell types, **Figure 3B** **, right**). The model simulations are based on an oxygen diffusion distance from the vessels estimated to be directly proportional to the distance at which hypoxia is detected away from blood vessels, measured experimentally from the HIF-1α staining to be 80±20 µm. The variation in this measurement leads to the variation in the simulated diameter. If the lymphoma is begun at sites within the lymph node other than the center (**Figure 4**), similar growth curves are computationally obtained as the whole node volume is eventually taken over by the proliferating tumor cells (results not shown). We note that since there is a distributed source of vessels in the tumor, the proliferation is relatively weakly sensitive to additional outside sources.

The tumor growth from the model calibrated from the cell-scale can be validated through theoretical analysis of the model based on previous mathematical and computational work [51], [54]–[56] (see **Text S1**). Assuming that the lymph node geometry is approximated by a 3D sphere, the model can be used to predict the tumor radius in time based on the ratio *A* of the rates of apoptosis to proliferation calculated from the experimental IHC data. The average ratio *A* = λ_A_/λ_M_ ∼0.4 for both drug-sensitive and drug-resistant cells. In comparison with the simulations based on the cell-scale calibration, this analysis predicts that the tumor would reach a diameter of ∼6 mm. Both the theoretical analysis and the tumor growth obtained through the simulations agree with the similar diameters observed experimentally *in vivo* (∼5 to 6 mm) (**Figure 7**).

### Simulation of diffusion barriers within the lymph node tumor

**Figure 8:**
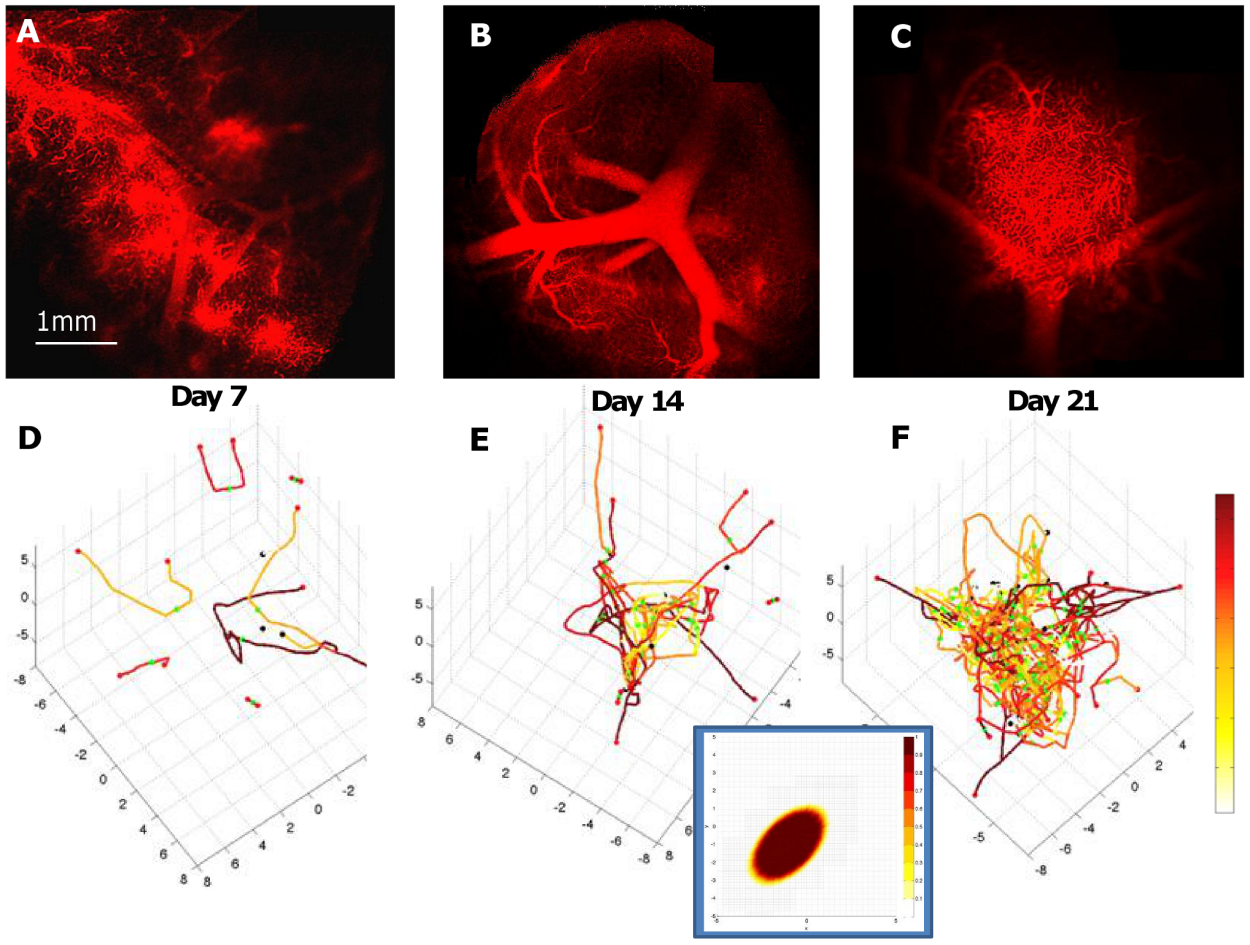
Vasculature and angiogenesis in the lymph node tumor. Observations in living mice using intravital microscopy (A, B, C: red – functional blood vessels; shown for *Eμ-myc p53-/-* tumor) provide information to qualitatively compare the vessel formation (D, E, F: red – highest flow; white – lowest; dots indicate vessel points of origin from pre-existing vasculature (not shown)) in the computational model (calibrated from other data, see **Text S1**). The modeling of diffusion of cell substrates (e.g., oxygen and cell nutrients) within the tumor enables prediction of the spatial distribution of lymphoma cells (inset, shown for one vessel cross-section; brown: highest concentration of cells; white: lowest concentration of cells) as their viability is modulated by access to the oxygen and nutrients diffusing from the vasculature into the surrounding tissue (**Eq. 2**).

In the model, simulations of the vasculature were qualitatively compared to independent intravital microscopy observations *in vivo* of a *Eμ-myc p53-/-* tumor in the same animal over time (**Figure 8**). The density of simulated viable tumor tissue (**Figure 3A** **, right**) as a function of the vascularization at day 21 qualitatively matches the density of the tissue observed experimentally (fraction of simulated viable cells in the 2D plane, >90% per mm^2^ in inset in **Figure 8**, vs. the average fraction of viable cells measured from H&E staining, 87%±6% per mm^2^), indicating that the overall vasculature function was modeled properly. The density of simulated endothelial tissue is also highest in the tumor core, as observed from histology. The increase in the lymphoma cell population disturbs the homogeneous distribution of cell substrates (such as oxygen and cell nutrients), leading to diffusion gradients of these substances that in turn affect the lymphoma cell viability. If the cell viability is established heterogeneously within the tumor, e.g., as observed experimentally in IHC with the *Eμ-myc Arf-/-* cells near the tumor periphery, the model predicts that the diffusion gradients would not be as pronounced. If the cell viability is higher near the center of the tumor, which is observed in IHC with the *Eμ-myc p53-/-* cells (**Figure 5**), then the gradients are predicted to be steeper and more uniform [28].

## Discussion

We integrate *in vivo* lymphoma data with computational modeling to develop a basic model of Non-Hodgkin's lymphoma. Through this work we seek a deeper quantitative understanding of the dynamics of lymphoma growth in the inguinal lymph node and associated physical transport barriers to effective treatment. We obtain histology data by very fine sectioning across whole lymph node tumors, thus providing detailed three-dimensional lymphoma information. We develop a computational model that is calibrated from these cell-scale data and show that the model can independently predict the tissue-scale tumor size observed *in vivo* without fitting to the data. We further show that this approach can shed insight into the tumor progression within the node, particularly regarding the physical reasons why some tumors might be resistant to drug treatment – a critical consideration when attempting to quantify and predict the treatment response. We envision that the modeling and functional relationships derived in this study could contribute with further development to patient-specific predictors of lymphoma growth and drug response.

Although the number of mice used for the experimental *in vivo* validation is limited, the model results are consistent with previous work. For example, a well-studied mechanism of physiological resistance is the dependence of cancer cell sensitivity to many chemotherapeutic agents on the proliferative state of the cell [28]. This physical mechanism is likely important in the difference in drug-sensitivity between the tumors formed from the two cell lines and will be explored in further studies. We found that the *Eμ-myc Arf-/-* cells tend to congregate at the periphery of the tumor (**Figure 5**), even though there are vessels in the interior of the tumor. This suggests the hypothesis that the more drug-sensitive *Eμ-myc Arf-/-* cells maintain better oxygenation at the expense of higher drug sensitivity by growing less compactly in the interior of the tumor – where there would be stronger competition for oxygen and cell nutrients – whereas the *Eμ-myc p53-/-* lymphoma cells may enhance their survival by closer packing in the core of the tumor. Cell packing density may present a barrier to effective drug penetration [57], which we have also modeled previously [28]. Closer packing could further increase the number of cells that would be quiescent due to depletion of oxygen and nutrients, as we specify in the model (**Materials and Methods**) and as we have simulated in previous work [28]. However, the proportion of chemoresistance inherent with *Eμ-myc p53-/-* that can be attributed to resistance at the genetic level compared to what can be attributed to suboptimal drug delivery and quiescence is unclear. In follow-up work we plan to measure drug amounts near various cells in order to begin answering this question, and to perform sensitivity analyses of the IC50 of each cell line with the computational model. This would provide a (model-generated) measure of how much of an effect suboptimal delivery could be attributed to resistance as compared to genetic effects (as measured by IC50).

Lymphoma cells are known to retain cell-cell adhesion, with strength associated with the lymphoma's originating cell type (B- or T-cell) [58]. Mechanisms of cell packing related to drug resistance may include weaker cell adhesion in *Eμ-myc Arf-/-* than in *Eμ-myc p53-/-* leading to higher cell density as well as a denser extra-cellular matrix in the latter [57]. Loss of ARF has been linked to increased cancer cell migration and invasion, and hence weaker cell-cell adhesion [59], associated with the binding of ARF to the transcriptional corepressor CtBP2 and promoting CtBP2 degradation [60]–[62].

Perhaps surprisingly, the experimental data indicate minimal presence of hypoxia within the tumor (**Figure 6B**). This may be due to the fact that lymphoma cells may associate with other cells including stromal cells in the tumor, and the consequent cytokine stimulation (e.g., IL-7) may also trigger proliferation [63]. We note that the oxygen diffusion length estimate is subject to variation, as calculated to be directly proportional to the hypoxic distances observed from the IHC; this may be improved by directly measuring the diffusing substances, e.g., oxygen. The simulated elastic tumor boundary may also introduce some variation into the size calculation. Nevertheless, even taking these variations into account, the model-calculated average ratio of apoptosis to proliferation, established from cell-scale measurements, implies that the tumor sizes fall within the range of the sizes estimated from the diameter measured with calipers *in vivo*. The hypothesis we test with the model by successful comparison to the experimental data is that the growth and eventual slowdown of these tumors is the balance of proliferation and death, which we have also previously observed for ductal carcinoma in situ [38]. Experimental evidence using bioluminescence imaging of living mice [53] demonstrates that lymphoma cells seed the tumor in the inguinal lymph node from other sites (e.g., spleen and bone marrow) in the mouse body at earlier times during the tumor growth. The model results are robust, however, because the tumor size by Day 21 predicted by the theory is independent of the earlier times; any influx of cells only provides an initial (transient) condition.

The staining also shows that apoptosis seems highest for drug-sensitive cells at the periphery of the tumor (Sections S1 and S5) compared to the center (Section S3) (both p-values = 0.04 using a Student's t-test with α = 0.05), and for drug-resistant cells it is highest in the more central regions (**Figure 6**). In accordance with biological observations [64], [65], [41], the model hypothesizes that increased hypoxia may lead to higher cell quiescence and hence drug resistance. In the experiments, angiogenesis is higher in the central regions, and is more pronounced for drug-resistant cells, suggesting that these cells are in a more angiogenic environment as a result of ongoing hypoxic stimulus. Higher tumor cell density around blood vessels suggests a functional relationship of cell viability as a function of nutrients, as we have implemented in the model (see **Materials and Methods**). However, apoptosis may not necessarily be driven solely by hypoxia, since lymphoma cells are known to have a cellular turnover rate that is on the order of days [66], [67]. We further note that angiogenesis is not necessarily triggered only by hypoxia. Lymphoma as well as stromal cells (such as tumor associated macrophages) may produce factors promoting angiogenesis (e.g., vascular endothelial growth factor or VEGF) under otherwise normoxic conditions.

The present work calibrates a computational model of lymphoma with experimental data from drug-sensitive and drug-resistant tumors. This data was derived from detailed IHC analysis of whole tumors, and validation of the model was performed via intravital microscopy measurements. The results suggest that differences in spatial localization of cells and vasculature, as well as in the transport phenomena in the tumor microenvironment may play a nontrivial role in the tumor behavior. This suggests that the genetic differences (*Eμ-myc Arf-/-* and *Eμ-myc p53-/-*) may provide a substantial compensation mechanism for these phenomena at the tissue scale in addition to the molecular as it relates to their drug resistance. We plan to verify this hypothesis in the future by assessing model predictions for therapeutic response of drug-sensitive and drug-resistant tumors in terms of cellular parameters such as proliferation, apoptosis, and hypoxia via both IHC and intravital microscopy.

## Supporting Information

Figure S1 **Example of calibration process of model parameters from the Ki-67 IHC data.** The proliferation parameter is calculated for both *Eμ-myc Arf-/-* (drug-sensitive) and *Eμ-myc p53-/-* (drug-resistant) lymphoma cells. This sample (from Set S3 in the center of the tumor) shows measurements obtained at the edge (periphery) and middle (center) of the section. Positive staining shown in the panels A–D is converted to red and negative staining to green in panels E–H to obtain a quantitative measure of proliferative activity, as calculated in the text. Results are shown in bottom right insets in panels E–H.
(TIF)

Figure S2 **Determination of optimal necrotic rate threshold for cell viability.** The necrosis rate is varied while the initial nutrient threshold is fixed at 0.5 to determine a range for which both the hypoxic fractions (A) and average apoptosis rate (B) match what is observed experimentally, finding that this range is from 5 to 7 (non-dimensionalized). We then varied the initial nutrient threshold while maintaining the necrosis rate invariant to confirm that the fraction of hypoxic cells (C) and average apoptosis rate (D) would remain within the experimentally observed ranges.
(TIF)

Table S1 **Range of key parameter values and corresponding baseline values for the computational model.** (M) values were calculated from the cell-scale immunohistochemistry data, (C) values were calibrated using these data, and (ND) are non-dimensionalized values.
(TIF)

Text S1 **Supplemental material.**
(DOC)
