# Supplementary figures and images for: An Integrated Computational/Experimental Model of Lymphoma Growth
Source: PLoS Comput Biol. 2013 Mar 28;9(3):e1003008. doi: 10.1371/journal.pcbi.1003008 (PMC3610621; doi:10.1371/journal.pcbi.1003008)

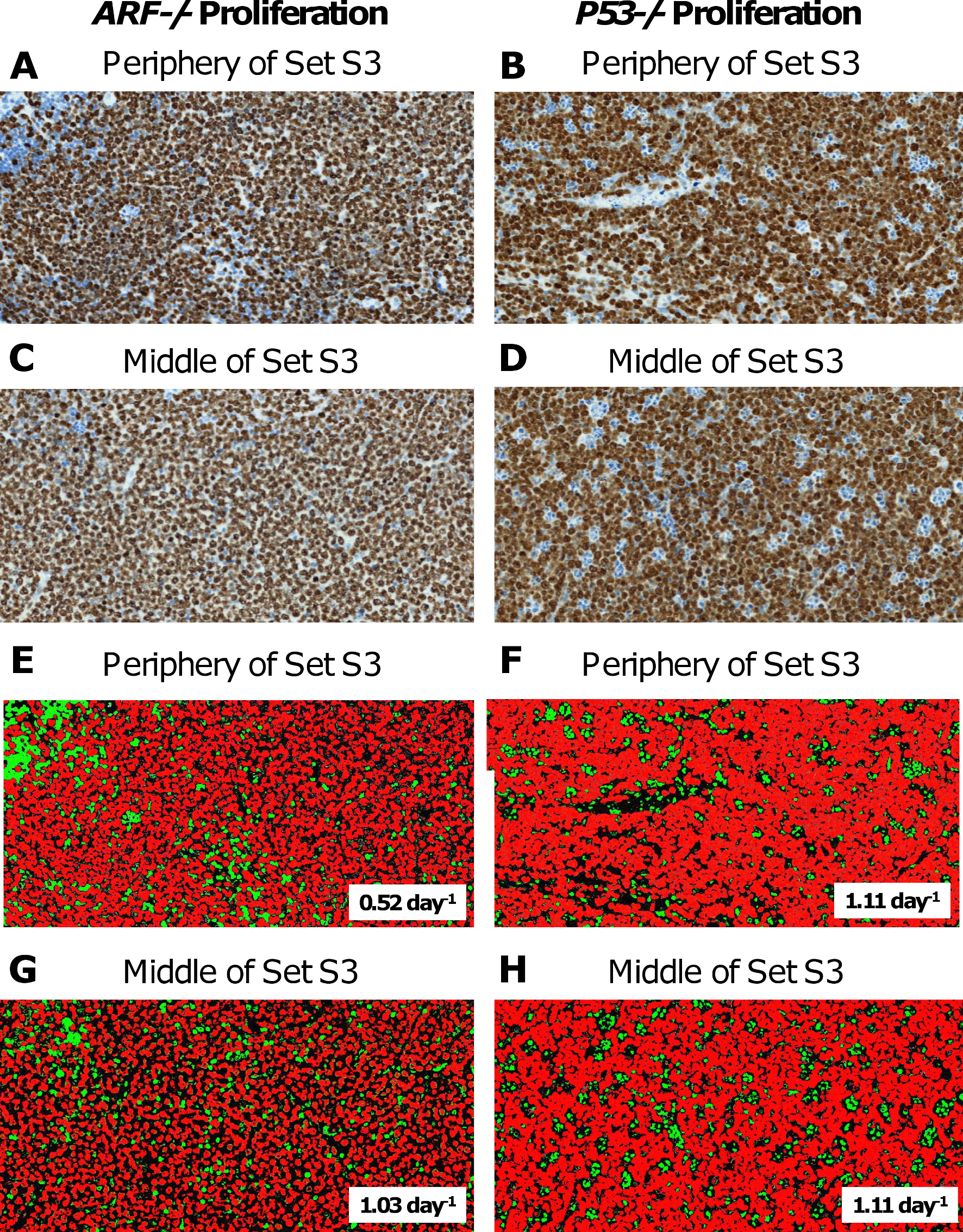

Supplement: Figure S1 — Example of calibration process of model parameters from the Ki-67 IHC data. The proliferation parameter is calculated for both Eμ-myc Arf-/- (drug-sensitive) and Eμ-myc p53-/- (drug-resistant) lymphoma cells. This sample (from Set S3 in the center of the tumor) shows measurements obtained at the edge (periphery) and middle (center) of the section. Positive staining shown in the panels A–D is converted to red and negative staining to green in panels E–H to obtain a quantitative measure of proliferative activity, as calculated in the text. Results are shown in bottom right insets in panels E–H. (TIF) [file pcbi.1003008.s001.tif]

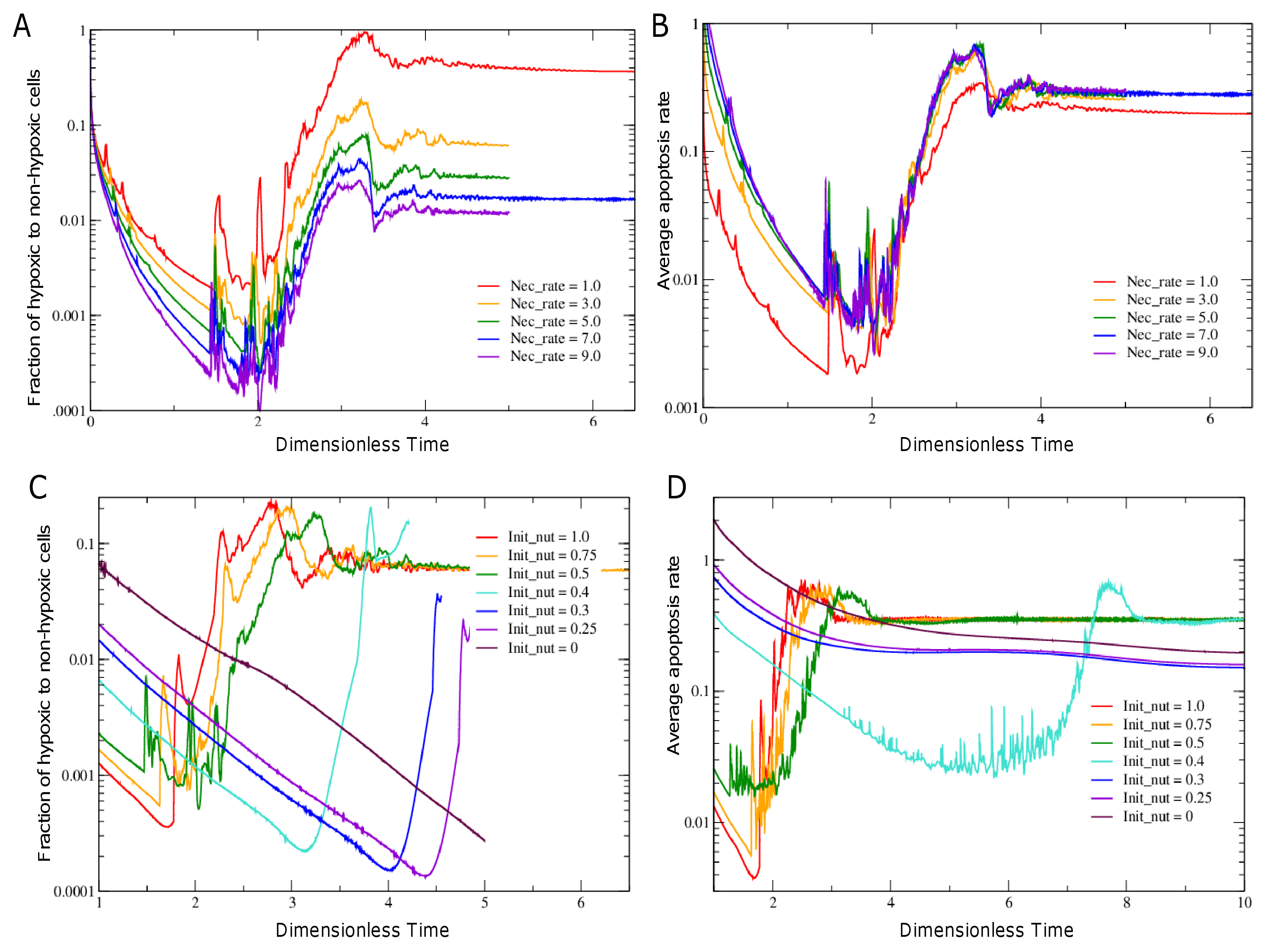

Supplement: Figure S2 — Determination of optimal necrotic rate threshold for cell viability. The necrosis rate is varied while the initial nutrient threshold is fixed at 0.5 to determine a range for which both the hypoxic fractions (A) and average apoptosis rate (B) match what is observed experimentally, finding that this range is from 5 to 7 (non-dimensionalized). We then varied the initial nutrient threshold while maintaining the necrosis rate invariant to confirm that the fraction of hypoxic cells (C) and average apoptosis rate (D) would remain within the experimentally observed ranges. (TIF) [file pcbi.1003008.s002.tif]

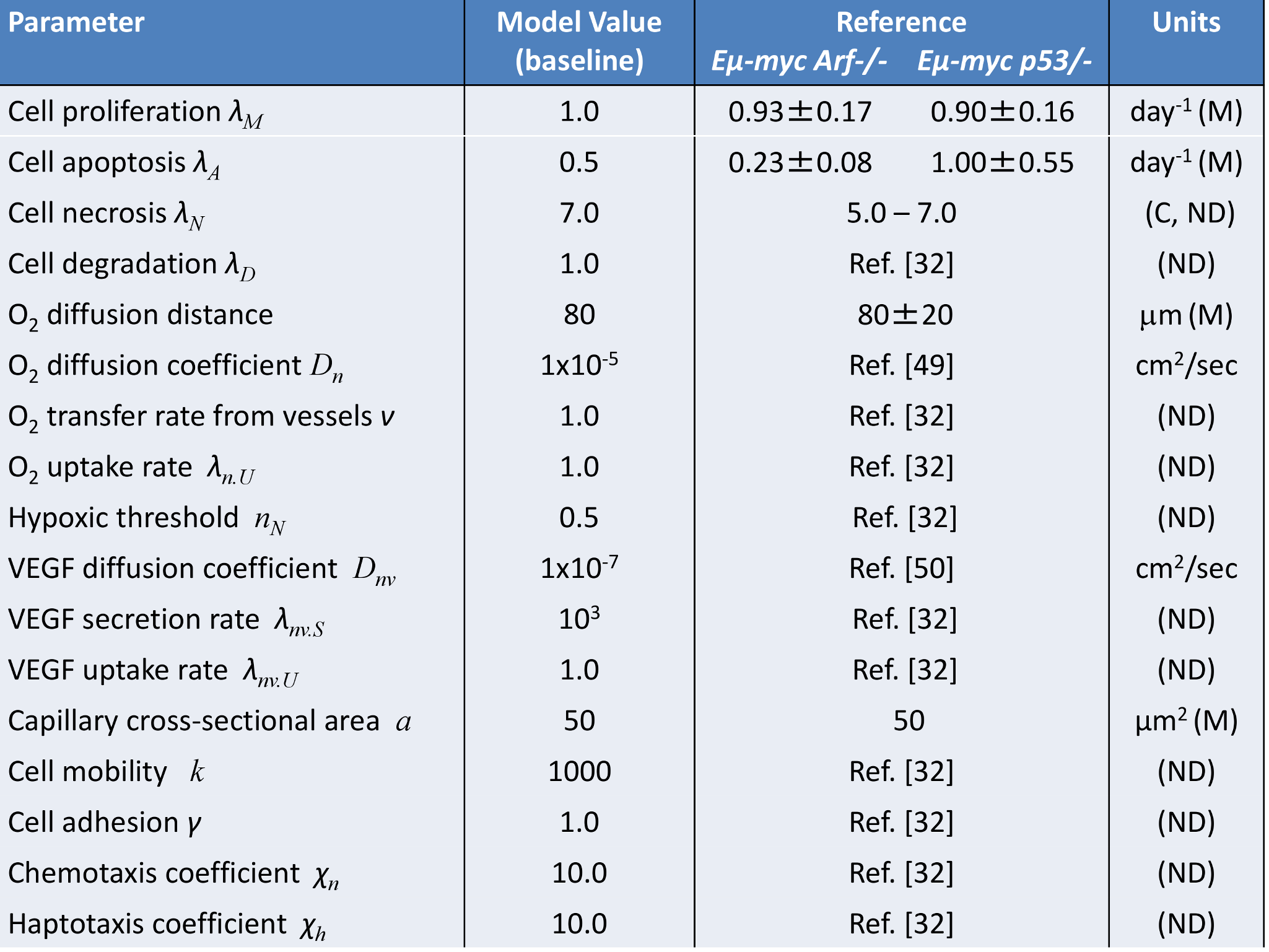

Supplement: Table S1 — Range of key parameter values and corresponding baseline values for the computational model. (M) values were calculated from the cell-scale immunohistochemistry data, (C) values were calibrated using these data, and (ND) are non-dimensionalized values. (TIF) [file pcbi.1003008.s003.tif]
