# Supplementary material for: An Integrated Computational/Experimental Model of Lymphoma Growth
Source: PLoS Comput Biol. 2013 Mar 28;9(3):e1003008. doi: 10.1371/journal.pcbi.1003008 (PMC3610621; doi:10.1371/journal.pcbi.1003008)
Supplement: Text S1 — Supplemental material. (DOC) [file pcbi.1003008.s004.doc]

**Text S1 (Supplemental Material)**

An Integrated Computational/Experimental Model of Lymphoma Growth

**Mathematical Model Description**

**Lymph Node Structure**

The inguinal lymph node is a small organ consisting of a fibrous capsule that surrounds and supports the cortex and medulla. Lymph fluid enters the node through afferent vessels that drain into the subcapsular sinus just inside the capsule. From there, lymph slowly filters through the cortex, into the medulla, collects in the medullary sinus, and exits through the efferent vessel. The cortex consists of multiple nodular, relatively dense follicles that primarily contain B-cell lymphocytes, each surrounded by more diffuse lymph tissue and supported by trabeculae extending from the outer capsule. The medulla primarily contains T-cell lymphocytes. The computational model simulates this initial structure as a round capsule with an afferent vessel on top and an efferent vessel at the bottom, thus collectively representing the group of afferent vessels (**Figure 4**). The cortex and medulla are given extra support by the extracellular matrix (ECM) and a network of reticular fibers to which lymphocytes adhere via integrins [68]; in the model these are collectively treated as a generic ECM affecting cell species chemotaxis and haptotaxis, as described in **Materials and Methods**.

Lymphoma is also well known for cell migration, both within the node and through the connecting lymph vessels [69,70]. Depending upon their immune activation, both B- and T-cells can migrate in amoeboid fashion [68,71] and exit the lymph node through the efferent vessel. This can be modeled by simulating distinct tumor cell populations with different cell migration capabilities [32]; for simplicity, we do not include this behavior in this initial implementation.

**Evolution of the Microvasculature**

In accordance with typical lymph node structure (**Figure 4A**), the pre-existing blood vessels reside mainly at the center of the lymph node, where tumor-induced vessels sprout. We approximate the pre-existing vasculature as a spherical region in the core of the lymph node, which serves as an initial and permanent nutrient source (see **Figure 4**). We treat the tumor neovasculature in 3D using a hybrid continuum-discrete, lattice-free random walk model of tumor angiogenesis, which refines earlier work by [46-48] (see below). The model generates a vascular topology based on tumor angiogenic regulators, represented by a continuum variable that reflects the excess of pro-angiogenic regulators compared to inhibitory factors [45]. Endothelial cells near the sprout tips proliferate and migrate by chemotaxis and haptotaxis [47,48] (see below). For simplicity, we track only the growth of leading endothelial cells, leaving the trails to form the capillary network. The vascular microarchitecture is captured via a set of rules; in particular, a leading endothelial cell has a fixed branching rate, while anastomosis occurs if a leading endothelial cell crosses a vessel trailing path. We apply recent advances [32] to track the aging of the vessels, which models their maturation as the endothelial cells tighten their junctions and become surrounded by basement membrane, as well as simulating flow within the vessel segments [72,73] (the simplified flow model excludes effects such as vessel adaptation, network remodeling, and feedback between tumor pressure and the vessels, which were developed in [72]). Parameter values for the vasculature are obtained from typical tumor measurements as detailed in [32,72]. For simplicity, in this initial implementation we assume that no oxygen or cell nutrients diffuse through the tumor boundary (i.e., no flux boundary condition).

**Tumor Angiogenesis Model**

As described in Frieboes et al., (2010) [32], vessels are generated based on Plank and Sleeman’s (2003, 2004) [47,48] biased circular random walk model. The trajectories of endothelial cells at the vessel tips trace a random walk characterized by circular paths. In 2D space, the vessel tip location (*x,y*) in time *t* can be characterized by its speed *s_tip_* and direction angle *θ*,

$\frac{\text{d}x}{\text{d}t}=s_{tip}\cos\theta,\text{ }\frac{\text{d}y}{\text{d}t}=s_{tip}\sin\theta.$ (5)

At each time step *k* the tip has a probability to continue in the same direction or to turn counterclockwise or clockwise by a small finite angle. This probability is generated through the normalized transition rate *τ*(*θ*),

$\tau(\theta,t)=\frac{1}{\sigma^{2}}\exp\left(2\int\frac{\mu}{\sigma^{2}}\text{d}\theta\right)$ (6)

where *μ* and *σ*^2^ are the expectation (mean) value and the variance of the turning rate, respectively, which can be adjusted according to the statistical analysis of the capillary network [32]. Both are functions of orientation *θ* and time *t*.

Chemotaxis and haptotaxis can be modeled as two taxis species by taking the mean turning rate *μ*, such that the tips tend to reorient themselves so that they are moving up the local gradient of the species [32]. The mean of the turning rate can be written as

$\mu=\sum_{i} -d_{i}\sin(\theta-\theta_{i}^{*}),$ (7)

where $\theta_{i}^{*}$is the preferred orientation along the gradient of each taxis species *i*, and *d_i_* is its corresponding turning coefficient which indicates the cell’s ability to reorient itself due to taxis, and is thus proportional to the magnitude of the gradient. Here, the preferred orientations $\theta_{chemotaxis}^{*}$and $\theta_{haptotaxix}^{*}$for the chemotaxis and haptotaxis species are the positive directions of the nutrient and fibronectin concentrations, respectively. Therefore,

$\mu=-d_{chemotaxis}\sin(\theta-\theta_{chemotaxis}^{*})-d_{haptotaxis}\sin(\theta-\theta_{haptotaxis}^{*}).$ (8)

The magnitude *s_tip_* of the tip cell velocity is also set proportional to the chemotaxis and haptotaxis gradients [32].

The model is easily extended to 3D by adding a second angle, *ϕ*, as the polar angle and using *θ* to represent the azimuthal angle. The tip location (*x,y,z*) is then tracked in time *t* by the following equations of motion:

(9)

where the transition rate of *ϕ* can be defined independently, similar to Eq.(6).

The local gradients of the taxis species are measured at each time step near the tip endothelial cells. The transition rate for each angle (*θ* and *ϕ*) is calculated with respect to the gradients, based on which it is determined if the cell stays at the current angle or turns counterclockwise or clockwise [32]. The new location of the cell is updated once the two angles are determined.

**Extra-cellular matrix (ECM) Density**

Vascular endothelial cells migrating through the extracellular matrix (ECM) release matrix-degrading enzymes (MDEs) that remodel and degrade the ECM. As the ECM is degraded, matrix macro-molecules such as fibronectin are released into the microenvironment, which are used as chemical factors in the haptotaxis (i.e., motion up gradients of matrix proteins) by endothelial tip cells moving towards tumor hypoxic regions. The change in time of the fibronectin concentration *f* can be modeled as described in (Frieboes et al., 2010) [32]:

(10)

where *R_f_* is a non-negative degradation rate constant, *m* is the MDE concentration, and *R_f,ves_* is a production rate constant by endothelial tip cells. *ϕ_tip_* represents the leading endothelial cells at the tip of the capillaries induced through angiogenesis. The MDEs are produced by endothelial cells and diffuse through the tissue before they decay. The equation governing this evolution is:

(11)

where *D_m_* is the diffusion coefficient, *R_m,ves_* is the MDE generating rate constant, and *R_m_* is a positive decay rate constant [32].

**Lymph Node Geometry**

We model the node as a surface *Γ* that is stretched by the growing tumor, with membrane normal velocity proportional to the proliferation-generated pressure gradient. We assume that the tissue surrounding the organ can be deformed sufficiently to accommodate expansion [74]. The geometry is described by a phase field variable *ψ* governed by a modified Cahn-Hilliard equation:

$\frac{\partial\psi}{\partial t}+\mathbf{v}\cdot\nabla\psi=\nabla\cdot\left(B\left(\psi\right)\nabla\mu_{\psi}\right),$ (12)

where $\psi(\mathbf{x},t)=\left\{\begin{matrix} \begin{matrix} 1 & \text{if }\mathbf{x}\in\Omega\end{matrix} \\ \begin{matrix} 0 & \text{if }\mathbf{x}\notin\Omega\end{matrix} \end{matrix}\right.$ specifies the position of the interface *Γ* through the narrow transition region characterized by a thickness parameter *ε* in the Cahn-Hilliard potential *μ_ψ_*. The function *B*(*ψ*) = 36 *ψ*^2^(1- *ψ*)^2^ specifies the interfacial region [75,52] where we want the Cahn-Hilliard potential to take effect. The velocity advecting *Γ* results from the mechanical pressure (*P*) generated by the proliferating tumor and the tissue surface tension [31]:

$\mathbf{v}=-\kappa\left(\nabla P-\frac{\gamma}{\varepsilon}\mu_{\psi}\nabla\psi\right),$ (13)

where *γ* specifies the strength of the surface tension, and *κ* is the tissue mobility in response to these exerted force. In reality, the tissue response to the mechanical pressure and the surface tension can be different. Here, we use the same mobility only for simplicity and without loss of generality, given that the actual units of pressure and surface tension can vary for different problems. For this study we do not let the geometry evolve during the tumor growth and only apply a negative surface tension to counterbalance the artificial surface tension produced by the Cahn-Hilliard term [76]. The geometry sets the effective domain boundary of tumor growth, where an equivalent no-flux boundary condition across the membrane should be imposed. To retain the solvability of the equations, we set the diffusion of substrates and the mobility of cells to an infinitesimal value instead of zero outside of the lymph node. For simplicity, a no-flux boundary condition is imposed on the computational domain boundaries.

**Velocity of Cell Species**

The constitutive laws for the mechanical fluxes ***J****_i_* and a generalized Darcy’s law for the cell velocities **u***_i_* are derived using the mixture energy *E* as described in (Wise et al., 2008 [31] and Lowengrub et al., 2010 [2]):

$E=\sum_{i=0}^{N} \int\Psi_{i}\text{d}x,$ (14)

where the Helmholtz free energy is

(15)

where *c*_1_*, . . . , c*_L_ are the concentrations of chemical and molecular species, *χ_il_* are taxis coefficients and *ε_il_* measures the strength of component interactions [31].

As derived in [31], thermodynamically consistent fluxes may be taken to be the generalized Fick’s law:

$1\leq i<N-1,$ (16)

and $J_{N}=-\sum_{i=1}^{N-1} J_{i}$, where $\overline{M}_{i}>0$ is a mobility, andare variational derivatives of the total energy *E* given by

$1\leq i<N.$ (17)

The velocities of the components may be determined in a manner that is mechanically and thermodynamically consistent [31]. Assuming that the solid and liquid volume fractions remain constant, and with constant in time and space, the resulting generalized Darcy laws for the velocities of the components are [31]:

(18)

$j\geq1,$ (19)

where *q* is the water pressure, *p* is the solid pressure and $\overline{k}_{0},\text{ }\overline{k},\text{ }\overline{k}_{j}$are positive definite motility matrices. The constitutive laws (16), (18) and (19) guarantee that in the absence of mass sources, the energy in equation (14) is non-increasing in time as the fields evolve [2].

The model may be simplified (as described in Wise et al., 2008 [31]) by assuming that tumor cells prefer to adhere to one another rather than to other cells (as observed experimentally [77]), and that no distinction is made between the adhesive properties of viable and dead cells. Accordingly, in equation (15), we may take where is the solid fraction of the tumor tissue and is the volume fraction of the host tissue (). Further, taking $\varepsilon_{ij}=0$ for *i, j < N* and $\varepsilon_{NN}=\overline{\varepsilon}$, the total adhesion energy (14) is:

(20)

The function *f* may be written as the difference of the following two convex functions [31]:

(21)

where one may take

and (22)

where *α*_1_ and *α*_2_ describe the strength of adhesion (attraction) of tumor cells to the host tissue and each other, respectively, and $\overline{E}$ is an overall energy scale. Setting *α*_1_ = *α*_2_, yields a double well energy *f* with minima at and and serves to define a well-delineated phase separation of the tumor () and the host tissues () [31].

From the flux constitutive equation (16) and the adhesion energy (20), the adhesion fluxes may be determined [2]. Since the densities of the components are matched, and taking the mobilities , where $\overline{M}$ is a positive constant, the fluxes are obtained [31]:

(23)

where *i* = 1*, . . . , N* − 1 and $J_{N}=J_{H}=-\sum_{i=1}^{N-1} J_{i},$, where it is used that the energy does not depend explicitly on *ϕ*_H_. The variational derivative is given by [31]:

(24)

Setting $\overline{k}_{i}=0$for *i >* 0, which is consistent with assuming the cells are tightly packed and that they move together with the mass-averaged velocity, the component velocities become [31]

$u_{0}=u_{W}=-\overline{k}_{W}\nabla q,$ (25)

(26)

where it is used that the energy does not explicitly depend on *ϕ*_0_ and *ϕ*_N_. In these equations, the terms dependent on *δE/δφ*_T_ represent the excess force due to adhesion and arise from cell–cell interactions. The coefficients $\overline{k}_{\text{W}}$, and $\overline{k}$ are motilities that reflect the response of the water and cells, respectively, to pressure gradients. These coefficients may depend on the volume fractions and other variables as the individual components may respond to the pressure and adhesive forces differently, but component mixtures tend to move together. The cell motilities describe the combined effects of cell–cell and cell–matrix adhesion. The constitutive choices (23), (25) and (26) guarantee that in the absence of mass sources (*S_i_* = 0), the adhesion energy is non-increasing as the fields evolve, while the total tumor mass is conserved [2].

**Theoretical Analysis (Simplified Model)**

As described in Lowengrub et al. (2010) [2], one can define Ω(*t*) to be the tumor domain, $\Sigma(t)$ to be the boundary between the tumor and host tissue, ***n*** to be the unit outward normal vector to $\Sigma$ and ***x*** to be the position in space. It may be assumed that the cell density is constant in the proliferating tumor domain (following [78-80,51]), which means that mass changes correspond to volume changes. Defining ***u*** to be the cell velocity, the local rate of volume change $\nabla\cdot u$is given by $\nabla\cdot u=\lambda_{P}$, where *λ*_p_ is the cell-proliferation rate and is given by *λ*_p_ = *bn* − *λ*_A_*,* where *n* denotes the concentration of a cell substrate (e.g. oxygen or glucose). The term *bn* is the rate of volume growth due to mitosis while *λ*_A_ is the rate of volume loss due to apoptosis. Thus, *b* is a measure of mitosis and *λ*_A_ is the rate of apoptosis (which may actually depend on *n*).

Cell substrates (e.g., oxygen and nutrients) diffuse through the ECM and are uptaken by tumor cells. Since the rate of diffusion of oxygen (or glucose) is much faster than the rate of cell proliferation, substrates may be regarded to be in a steady state for a given tumor volume (e.g., [78-80,51]). Therefore,

$0=D\nabla^{2}n+\Gamma$ (27)

where $\Gamma$ is the rate at which substrates are added to Ω and is given by

$\Gamma=\lambda_{B}(n_{B}-n)-\lambda n$*,*  (28)

where the first term describes the source of substrates from the vasculature while the second describes substrate uptake by cells. Here, *λ*_B_ is the blood-tissue transfer rate of nutrient, *n*_B_ is the concentration of substrates in the blood, and *λ* is the rate of consumption of substrates by tumor cells. In this simplified model, the vasculature is assumed to be uniform and thus vascular growth is associated with a bulk source of oxygen, nutrients and growth factors.

To determine the cell velocity, Darcy’s law may be used as the constitutive assumption

(e.g. [79-81,51]):

$u=-\mu\nabla P$, (29)

where *P* is the oncotic (solid) pressure and *μ* is a mobility that reflects the combined effects of cell–cell and cell–matrix adhesion.

The boundary conditions on the tumor interface $\Sigma$ may be taken to be [2]:

$(n)_{\Sigma}=n^{\infty},$ (30)

$(P)_{\Sigma}=\gamma\kappa,$ (31)

where the pressure boundary condition (31) reflects the influence of cell–cell adhesion through the parameter *γ* and *κ* is the local total curvature. For simplicity, we assume *n*^∞^ is constant so that the substrates are uniform outside the tumor.

The normal velocity $V=n\cdot(u)_{\Sigma}$of the tumor boundary is [2]:

$V=-\mu n\cdot(\nabla P)_{\Sigma}$*.*  (32)

Following [79-81,51] and others, we assume that *λ*, *λ*_A_, *λ*_B_, *n*_B_, *b* are uniform. Following [51], we denote *λ*_M_ = *bn*^∞^ to be the characteristic mitosis rate, $\lambda_{R}=\mu\gamma L_{D}^{-3}$to be the intrinsic relaxation time scale, and $B=n_{B}\lambda_{B}/n\infty(\lambda_{B}+\lambda)$to be a measure of the extent of vascularization. Introducing the non-dimensional length scale $L_{D}=D^{1/2}(\lambda_{B}+\lambda)^{-1/2}$, and time scale $\lambda_{R}^{-1}$ , a modified concentration $\overline{\Gamma}$ and pressure $\overline{p}$ can be defined such that [51]:

$n=n^{\infty}(1-(1-B)(1-\overline{\Gamma}))$ (33)

$P=\frac{\gamma}{L_{D}}\left(\overline{p}+(1-\overline{\Gamma})G+AG\frac{x\cdot x}{2d}\right)$*,*  (34)

where *G* and *A* measure the relative strength of cell–cell and cell–matrix adhesion and apoptosis, respectively [51]:

$G=\frac{\lambda_{M}}{\lambda_{R}}(1-B)$ (35)

$A=\frac{\lambda_{A}/\lambda_{M}-B}{1-B}$ (36)

The non-dimensional equations for $\Gamma$ and *p* then are [2]:

$\nabla^{2}\Gamma-\Gamma=0$*,*  (37)

$\nabla^{2}p=0$*,* (38)

with boundary conditions:

$(\Gamma)_{\Sigma}=1$*,*  (39)

$(p)_{\Sigma}=\kappa-AG\frac{(x\cdot x)_{\Sigma}}{2d}$ (40)

in a tumor of *d*-dimension (*d* = 2, 3). The non-dimensional normal velocity of the tumor–host interface is [51]:

$V=-n\cdot(\nabla p)_{\Sigma}+Gn\cdot(\nabla\Gamma)_{\Sigma}-AG\frac{n\cdot(x)_{\Sigma}}{2d}$ (41)

**Text S1 References**

2. Lowengrub JS, Frieboes HB, Jin F, Chuang YL, Li X, et al. (2010) Nonlinear modelling of cancer: bridging the gap between cells and tumours. Nonlinearity 23: R1-R9.

31. Wise SM, Lowengrub J, Frieboes HB, Cristini V (2008) Three-dimensional multispecies nonlinear tumor growth--I Model and numerical method. J Theor Biol 253: 524-543.

32. Frieboes HB, Jin F, Chuang YL, Wise SM, Lowengrub JS, et al. (2010) Three-dimensional multispecies tumor growth-II: Tumor invasion and angiogenesis. J Theor Biol 264: 1254-1278.

45. Ruan J, Hajjar K, Rafii S, Leonard JP (2009) Angiogenesis and antiangiogenic therapy in non-Hodgkin’s lymphoma. Annals of Oncology 20: 413–424.

46. Anderson AR, Chaplain MA (1998) Continuous and discrete mathematical models of tumor-induced angiogenesis. Bull Math Biol 60: 857-899.

47. Plank MJ, Sleeman BD (2003) A reinforced random walk model of tumour angiogenesis and anti-angiogenic strategies. Math Med Biol 20: 135-181.

48. Plank MJ, Sleeman BD (2004) Lattice and non-lattice models of tumour angiogenesis. Bull Math Biol 66: 1785-1819.

51. Cristini V, Lowengrub J, and Nie Q (2003) Nonlinear simulation of tumor growth. J Math Biol 46: 191-224.

52. Li X, Lowengub J, Rätz A, Voigt A (2009) Solving PDEs in complex geometries: a diffuse domain approach. Commun Math Sci 7: 81-107.

68. Kaldjian EP, Gretz JE, Anderson AO, Shi Y, Shaw S (2001) Spatial and molecular organization of lymph node T cell cortex: a labyrinthine cavity bounded by an epithelium-like monolayer of fibroblastic reticular cells anchored to basement membrane-like extracellular matrix. Int Immunol 13: 1243-1253.

69. Tavor S, Petit I, Porozov S, Goichberg P, Avigdor A, et al. (2005) Motility, proliferation, and egress to the circulation of human AML cells are elastase dependent in NOD/SCID chimeric mice. Blood 106: 2120-2127.

70. Crainie M, Belch AR, Mant MJ, Pilarski LM (1999) Overexpression of the receptor for hyaluronan-mediated motility (RHAMM) characterizes the malignant clone in multiple myeloma: identification of three distinct RHAMM variants. Blood 93: 1684-1696.

71. Chang TW, Celis E, Eisen HN, Solomon F (1979) Crawling movements of lymphocytes on and beneath fibroblasts in culture. Proc Natl Acad Sci USA 76: 2917-2921.

72. Macklin P, McDougall S, Anderson AR, Chaplain MA, Cristini V, et al. (2009) Multiscale modelling and nonlinear simulation of vascular tumour growth. J Math Biol 58: 765-798.

73. Wu M, Frieboes HB, McDougall S, Chaplain MA, Cristini V, et al. (2013) The effect of interstitial pressure on tumor growth: coupling with the blood and lymphatic vascular systems. J Theor Biol 320: 131-151.

74. Peckham MJ. (1973) Quantitative cytology and cytochemistry of Hodgkin’s tissue labelled in vivo with tritiated thymidine. Br J Cancer 28: 332-339.

75. Rätz A,Voigt A (2005) Edge Diffusion in Phase-Field Models for Epitaxial Growth. Int Series Num Math 149: 115-125.

76. Sun Y, Beckermann C (2007) Sharp Interface Tracking Using the Phase-Field Equation. J Comp Phys 220: 626-653.

77. Armstrong PB (1971) Light and electron microscope studies of cell sorting in combinations of chick embryo and neural retina and retinal pigment epithelium. Wilhelm Roux Arch. 168: 125–141.

78. Greenspan HP (1976) On the growth and stability of cell cultures and solid tumors. J Theor Biol 56: 229–242.

79. Byrne HM, Chaplain MAJ (1995) Growth of nonnecrotic tumors in the presence and absence of inhibitors. Math Biosci 130: 151–181.

80. Friedman A, Reitich F (1999) Analysis of a mathematical model for the growth of tumors. J Math Biol 38: 262–284.

81. Greenspan HP (1972) Models for the growth of a solid tumor by diffusion. Stud Appl Math 51: 317–340.
